# Human DEF6 deficiency underlies an immunodeficiency syndrome with systemic autoimmunity and aberrant CTLA-4 homeostasis

**DOI:** 10.1038/s41467-019-10812-x

**Published:** 2019-07-15

**Authors:** Nina K. Serwas, Birgit Hoeger, Rico C. Ardy, Sigrun V. Stulz, Zhenhua Sui, Nima Memaran, Marie Meeths, Ana Krolo, Özlem Yüce Petronczki, Laurène Pfajfer, Tie Z. Hou, Neil Halliday, Elisangela Santos-Valente, Artem Kalinichenko, Alan Kennedy, Emily M. Mace, Malini Mukherjee, Bianca Tesi, Anna Schrempf, Winfried F. Pickl, Joanna I. Loizou, Renate Kain, Bettina Bidmon-Fliegenschnee, Jean-Nicolas Schickel, Salomé Glauzy, Jakob Huemer, Wojciech Garncarz, Elisabeth Salzer, Iro Pierides, Ivan Bilic, Jens Thiel, Peter Priftakis, Pinaki P. Banerjee, Elisabeth Förster-Waldl, David Medgyesi, Wolf-Dietrich Huber, Jordan S. Orange, Eric Meffre, David M. Sansom, Yenan T. Bryceson, Amnon Altman, Kaan Boztug

**Affiliations:** 1 Ludwig Boltzmann Institute for Rare and Undiagnosed Diseases, Vienna, Austria; 2 CeMM Research Center for Molecular Medicine of the Austrian Academy of Sciences, Vienna, Austria; 3 St. Anna Children’s Cancer Research Institute (CCRI), Vienna, Austria; 4 Centre for Hematology and Regenerative Medicine, Department of Medicine Huddinge, Karolinska Institutet, Karolinska University Hospital Huddinge, Stockholm, Sweden; 5 Division of Cell Biology, La Jolla Institute for Allergy & Immunology, La Jolla, CA 92037 USA; 6 Department of Pediatrics and Adolescent Medicine, Medical University of Vienna, Vienna, Austria; 7 Childhood Cancer Research Unit, Department of Women’s and Children’s Health, Karolinska Institutet, Karolinska University Hospital Solna, Stockholm, Sweden; 8 Clinical Genetics Unit, Department of Molecular Medicine and Surgery, and Center for Molecular Medicine, Karolinska Institutet, Karolinska University Hospital Solna, Stockholm, Sweden; 9 Center for Pathophysiology of Toulouse Purpan, INSERM UMR1043, CNRS UMR5282, Paul Sabatier University, Toulouse, France; 10 Institute of Immunity and Transplantation, Division of Infection & Immunity, School of Life and Medical Sciences, University College London, Royal Free Hospital, Rowland Hill Street, London, NW3 2PF UK; 11 Department of Pediatrics, Baylor College of Medicine and Center for Human Immunobiology, Texas Children’s Hospital, Houston, TX 77030 USA; 12 Institute of Immunology, Center for Pathophysiology, Infectiology and Immunology, Medical University of Vienna, Vienna, Austria; 13 Clinical Institute of Pathology, Medical University of Vienna, Vienna, Austria; 14 Department of Immunobiology, Yale University School of Medicine, New Haven, CT 06511 USA; 15 Department of Rheumatology and Clinical Immunology, University Medical Center Freiburg, Freiburg, 79106 Germany; 16 Astrid Lindgren Children’s Hospital, Karolinska University Hospital Huddinge, Stockholm, Sweden; 17 Department of Pediatrics and Adolescent Medicine, Division of Neonatology, Pediatric Intensive Care and Neuropediatrics, Medical University of Vienna, Vienna, Austria; 18 St. Anna Kinderspital, Department of Pediatrics and Adolescent Medicine, Medical University of Vienna, Vienna, Austria; 19 Present Address: Department of Pathology, University of California San Francisco, San Francisco, CA USA; 20 Present Address: Centre for Paediatrics and Adoloscent Medicine, Hannover Medical School, Hannover, Germany; 21 Present Address: Columbia University Medical Center, Columbia, NY USA; 22 Present Address: Takeda (Shire), Vienna, Austria; 23 Present Address: MD Anderson Cancer Center, Houston, TX USA

**Keywords:** Genetics, Immunology, Diseases

## Abstract

Immune responses need to be controlled tightly to prevent autoimmune diseases, yet underlying molecular mechanisms remain partially understood. Here, we identify biallelic mutations in three patients from two unrelated families in *differentially expressed in FDCP6 homolog (DEF6)* as the molecular cause of an inborn error of immunity with systemic autoimmunity. Patient T cells exhibit impaired regulation of CTLA-4 surface trafficking associated with reduced functional CTLA-4 availability, which is replicated in *DEF6*-knockout Jurkat cells. Mechanistically, we identify the small GTPase RAB11 as an interactor of the guanine nucleotide exchange factor DEF6, and find disrupted binding of mutant DEF6 to RAB11 as well as reduced RAB11^+^CTLA-4^+^ vesicles in *DEF6*-mutated cells. One of the patients has been treated with CTLA-4-Ig and achieved sustained remission. Collectively, we uncover DEF6 as player in immune homeostasis ensuring availability of the checkpoint protein CTLA-4 at T-cell surface, identifying a potential target for autoimmune and/or cancer therapy.

## Abstract (web summary)

CTLA-4 is critical for balancing protective immunity with self-tolerance. Here the authors identify homozygous *DEF6* mutations in patients with severe autoimmunity, one of which received and responds to CTLA-4-Ig, and show that DEF6 is crucial for CTLA-4 cell surface trafficking and immune regulatory function.

## Introduction

Although immune dysregulation and autoimmunity are hallmarks of multiple human diseases, their underlying molecular pathological mechanisms remain poorly understood^1^. Studying monogenic disorders with predominant autoimmunity offers an attractive strategy to identify core regulators of immune homeostasis^2^. Key regulatory components which help tune immune responses include regulatory T cells (T_regs_)^3^ and the checkpoint protein CTLA-4^4^. CTLA-4 is constitutively expressed on T_regs_ and upon activation on activated conventional helper-T cells (T_conv_). CTLA-4 ligand engagement results in rapid internalization from cell surfaces by clathrin-mediated endocytosis, and shuttling to either lysosomes or RAB11^+^ recycling endosomes^5–8^. Functionally, CTLA-4 competes with the activating co-receptor CD28 for interaction with their shared ligands CD80/CD86 expressed on antigen-presenting cells (APCs)^9^, thereby inhibiting T-cell costimulation. Binding of CTLA-4 to CD80/CD86 results in ligand transendocytosis into T cells, sequestering the costimulatory ligands from APCs. Inside the T cell, CD80/86 are guided to lysosomal degradation^10^. Interaction of CTLA-4 with lipopolysaccharide-responsive and beige-like anchor protein (LRBA) is essential for prevention of its own degradation^11^. The importance of CTLA-4 in regulating human immune tolerance is underlined by several SNPs conferring increased risk of autoimmunity^12^ and further solidified with the recent identification of patients with monoallelic mutations in *CTLA4* or biallelic mutations in *LRBA* suffering from severe autoimmunity^13–18^. Additional cellular regulators of CTLA-4 and their relevance to human disease remain to be investigated.

DEF6, also known as IRF4 binding protein (IBP)^19^ or SWAP-70-like adaptor of T cells (SLAT)^20^ is a unique guanine nucleotide exchange factor (GEF) which has an inverse conformation of the PH-DH domain compared to conventional GEFs^21^. DEF6 acts downstream of the T-cell receptor (TCR) and can be phosphorylated by the tyrosine-protein kinases LCK^21^ and ITK^22^. It can activate small GTPases of the RHOA^21^ and Ras family^23^, promoting Ca^2+^ signaling, NFAT1 activation^24^, and T-cell adhesion^23^. Additionally, DEF6 binds and negatively regulates the transcription factor IRF4^25,26^. Murine knockout studies have illustrated a role of Def6 in immunological synapse formation^27^, Th1/Th2 lineage differentiation^24^, IL17 and IL21 production^26^, bacterial phagocytosis^28^, T-cell proliferation^29^, as well as a possible role in early-onset large vessel vasculitis^26^ and autoimmunity^27^. Interestingly, other studies of *Def6*-knockout mice contrarily revealed resistance to uveitis and experimental autoimmune encephalitis^30,31^, and to date it remains unclear whether susceptibility to autoimmunity is dependent on the genetic background of the mice or other factors. Thus, the role of DEF6 in autoimmunity has remained controversial and partially enigmatic.

Here, we uncover an inborn error of immunity caused by biallelic mutations in *DEF6* and characterized by early-onset systemic autoimmunity. We find impaired CTLA-4 availability and trafficking, due to decreased interaction of mutated DEF6 with the small GTPase RAB11, as the mechanistic basis for the autoimmune manifestations.

## Results

### Systemic autoimmunity in three patients from two families

**Fig. 1:**
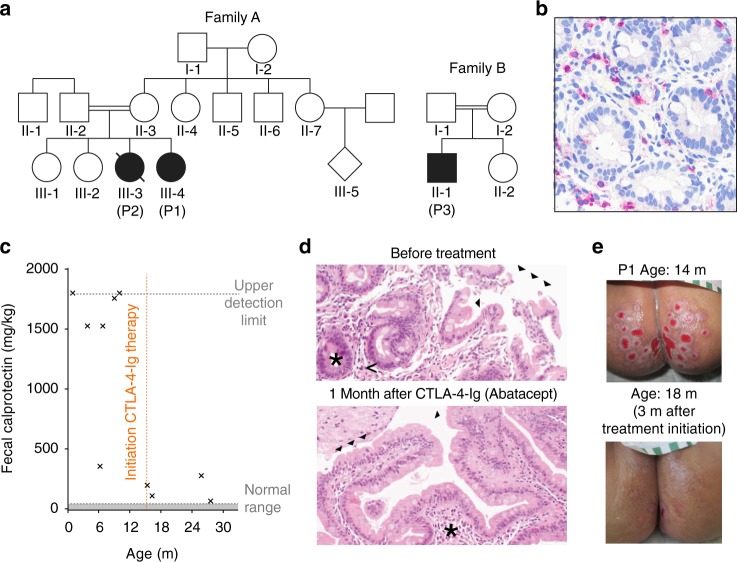
Systemic autoimmunity in three patients from two families. **a** Pedigree of families A and B. Filled symbols – affected patients (P). **b** Colon biopsy of P1 reveals T-cell infiltration (red: anti-CD3). **c** Fecal calprotectin values reveal therapy-dependent reduction of bowel inflammation in P1. **d** Duodenal biopsies at the age of 5 months (top) showed incomplete villous atrophy with villi focally reduced and plump (closed arrows). The inflammatory infiltrate contains clusters of eosinophilic granulocytes (lined arrows) and only few crypts with isolated apoptotic figures (asterisk). At the age of 16 months (bottom, 1 month of therapy with Abatacept, see Fig. S1e) duodenal biopsies showed presence of villi (closed arrows) and no signs of acute inflammation in the lamina propria (asterisk) of P1. **e** Perianal fissures of P1 before (top) and after (bottom) therapy initiation present a marked improvement of patient quality of life (m - months)

We studied three patients with severe autoimmune manifestations. Patient 1 is female (P1, Family A) born to consanguineous Pakistani parents (Fig. 1a) who presented with severe watery diarrhea in the first month of life. Endoscopy revealed atrophy of gastric mucosa and villous atrophy with pronounced T- and eosinophilic cell infiltration in the colon and duodenum (Fig. 1b and Fig. S1a). Further disease features included hepatosplenomegaly, dilated cardiomyopathy, and increased susceptibility to viral and bacterial infections suggesting a primary immune defect (Tables 1 and 2). Immune phenotyping revealed reduced CD8^+^ T-cell numbers (Table 1) and slightly reduced percentages of CD25^high^CD127^low^FOXP3^+^ T_regs_ (Fig. S1b) in the circulation. Immunoglobulin levels were not consistently altered (Table 1), only few CD19^+^CD27^+^IgD^−^ class-switched B cells were detected (Fig. S1c), and specific antibody responses were impaired (Table 2). Clinical signs of autoimmunity were paralleled by detectable anti-neutrophil cytoplasmic antibodies (ANCA) and autoantibodies against cardiolipin, smooth muscle protein, and β2-glycoprotein (Table 2). NK cells were in the normal range, and neutrophil function including oxidative burst as well as phagocytosis of opsonized bacteria was not impaired (Table 2). A serum cytokine/chemokine blot did not reveal elevation of pro-inflammatory cytokines but rather reduced levels of serum IL-12 and IL-6 compared to a healthy control (Fig. S1d). Upon clinical deterioration of symptoms, we initiated CTLA-4-Ig (Abatacept) treatment at 4-weekly intervals starting at 15 months of age (Fig. S1e). Consequently, bowel inflammation decreased markedly as reflected by fecal calprotectin values (Fig. 1c). Lymphocytic infiltration and complete villous atrophy of the duodenum improved within one month of treatment (Fig. 1d). In addition, persisting perianal lesions reversed and did not recur (Fig. 1e). P1 was consequently discharged and treated as an outpatient (Fig. S1e). To date, ~4 years after treatment initiation, no overt signs of autoimmunity have reoccurred, and cardiorespiratory fitness has been stable without arrhythmias or other overt pathology. Regular immunoglobulin treatment is given. Recurrent infections requiring antibiotic treatment have persisted (Fig. S1e). The female sibling of P1 (patient 2 or P2) had been diagnosed earlier with a systemic autoimmune/autoinflammatory disease that included bowel inflammation, hepatomegaly, cholestasis, and cardiac ventricular septal defect. P2 also presented with recurrent infections and exhibited reduced numbers of lymphoid cells (Table 1, Table 2), however immunological investigations could not be performed in-depth since P2 died at 10.5 months of age due to cardiomyopathy-associated cardiac and multi-organ failure.

**Table 1:** Immunological data on patients with *DEF6* mutations

Patient (age)	P1 (1–4 m)	P1 (5–8 m)	P1 (9–10 m)	P2 (4 m)	P2 (5–6 m)	P2 (7 m)	P3 (7–13 m)	P3 (5 y 3 m)	P3 (5 y 7 m)
ALC (cells/mm³) (normal range)	6040 (4054–7048)		6250 (3320–7006)	**2230** (3320–7006)	**1450** (3320–7006)	**1930** (3320–7006)	**1904** (3873–6141)	**1779** (2340–5028)	**1753** (2340–5020)
*Lymphocyte subsets*									
CD3^+^ (%)	77 (62.7–81.6)		68 (51.8–74.2)	62 (51.8–74.2)	**78** (51.8–74.2)	67 (51.8–74.2)	**60** (60.7–75.8)	70 (59.7–77.6)	72 (59–7–77–6)
(cells/mm³)	4650 (3180–5401)		4250 (2284–4776)	**1380** (2284–4776)	**1130** (2284–4776)	**1290** (2284–4776)	**1137** (2542–4933)	**1265** (1578–3707)	**1263** (1578–3707)
CD4^+^ (%)	65 (42.8–65.7)		53 (34.9–53.1)	**56** (34.9–53.1)	**67** (34.9–53.1)	**61** (34.9–53.1)	34 (35.0–51.9)	35 (31.1–47.4)	34 (31.1–47.4)
(cells/mm³)	**3930** (2330–3617)		3310 (2284–4776)	**1250** (2284–4776)	**970** (2284–4776)	**1170** (2284–4776)	**653** (1573–2949)	**635** (870–2144)	**594** (870–2144)
CD8^+^ (%)	**11** (15–23)		**11** (12.8–27.1)	**7** (12.8–27.1)	**8** (12.8–27.1)	**8** (12.8–27.1)	18 (16.1–29.4)	28 (16.0–26.9)	**34** (16.0–26.9)
(cells/mm³)	**660** (712–1361)		690 (524–1583)	**160** (524–1583)	**120** (524–1583)	**150** (524–1583)	**351** (656–1432)	513 (472–1107)	592 (472–1107)
CD19^+^ (%)	8 (7.4–21.3)		19 (17–37.2)	18 (17–37.2)	**12** (17–37.2)	18 (17–37.2)	21 (14.3–28.2)	16 (12.9–29.2)	14 (12.9–29.2)
(cells/mm³)	480 (315–1383)		1190 (776–2238)	**400** (776–2238)	**170** (776–2238)	**350** (776–2238)	**415** (733–1388)	**281** (434–1274)	**238** (434–1274)
CD16^+^56^+^ (%)	12 (4.2–14.8)		12 (4–15.1)	15 (4–15.1)	11 (4–15.1)	18 (4–15.1)	9 (4.0–13.8)	7 (4.7–16.2)	8 (4.7–16.2)
(cells/mm³)	730 (201–870)		750 (230–801)	330 (230–801)	**160** (230–801)	350 (230–801)	**183** (186–724)	**129** (155–565)	**143** (155–565)
CD3^+^CD45RA^+^ (cells/mm³)	78 3630		82 3490			63 810	54 580	43 553	39 496
CD3^+^CD45RO^+^ (cells/mm³)	6 280		9 380			14 180	36 409	57 721	60 767
CD3^+^TCRαβ^+^ (cells/mm³)	75 4530		62 3880			62 1190	99 1115	96 1214	88 1118
CD3^+^TCRγδ^+^ (cells/mm³)	2 (0.7–4.1) 120		**2** (2.8–5.8) 130			**2** (2.8–5.8) 40	1 17	4 68	11 145
*Immunoglobulins*									
IgG (g/L)	**1.60** (↓) (4–9.8)	4.32	9.59	5.60	5.51		6.9		
IgA (g/L)	**0.009 (↓)** (0.17–0.94)	0.69	**2.34** (↑)	0.42	**1.09** (↑)		0.25		
IgM (g/L)	**0.17 (↓)** (0.34–2.1)	0.91	**4.74** (↑)	0.67	**2.92** (↑)		0.45		

Lymphocyte reference values (in brackets) were taken from ref. ^68^. Values outside reference range are marked in bold. Immunoglobulin (Ig) concentration was tested at least 4 weeks after the last intravenous Ig treatment. P1 was vaccinated three times with Prevenar 13® (Pfizer: pneumococcal polysaccharide conjugated vaccine) and INFANRIX hexa® (GlaxoSmithKline: *Corynebacterium diphtheriae*, *Clostridium tetani*, *Bordetella pertussis*, *Haemophilus influenzae type 1B*, hepatitis B virus, poliovirus) at the age of 3, 4, and 10 months. The higher values might be caused by the presence of maternal antibodies

m months, y years, ALC absolute lymphocyte count, TCR T-cell receptor, Ig Immunoglobulin

**Table 2:** Clinical characteristics of patients with *DEF6* mutations

Patient (age)	P1	P1 (1–4 m)	P1 (5–8 m)	P1 (9–10 m)	P1 (11–12 m)	P2	P3
Neutrophil function							
Phagocytosis	normal (*E. coli opson*.,						
	*S. pneumoniae opson.)*						
Oxidative burst	normal						
Hemoglobin							54 g/L (**↓**) (2 y 3 m)
Vaccination response							
*C. tetani*		0.73 IU/ml (>=0.4 IU/ml)	**0.06** IU/ml (>=0.4 IU/ml)	**0.05** IU/ml (>=0.4 IU/ml)			
*C. diphtheriae*		**0.05** IU/ml (>=0.4 IU/ml)	**0.02** IU/ml (>=0.4 IU/ml)	**0.02** IU/ml (>=0.4 IU/ml)			
*S. pneumonia*		**1:76** (>=1:200)	**1:20** (>=1:200)	**1:20** (>=1:200)			
*H. influenzae*		**0.76** µg/ml (>=1 µg/ml)	**0.06** µg/ml (>=1 µg/ml)	**0.07** µg/ml (>=1 µg/ml)			
*B. pertussis*			**0.6** VE (>=11 VE)				
Autoantibodies							
ANCA				Positive (1:160)	Positive (1:40)		
Cardiolipin (IgG)				n.d.	Positive (12.1 U/ml)		
Beta2-glycoprotein (IgG) (IgM)				Elevated (10.5 U/ml) Normal (4.9 U/ml)	Positive (28.8 U/ml) Positive (8 U/ml)		
Direct Coombs test							Positive
Recurrent infections							
Bacteria	*S. pneumoniae, S. aureus, S. epidermis, E. aerogenes, E. cloacae, E. faecalis*					*E. aerogenes, K. oxytoca, S. epidermis, E. faecalis*	
Virus	Rhinovirus, influenza B, respiratory syncytial virus, rotavirus					Not specified	
Fungi	Not specified					Malassezia furfur	

Reference values in brackets. Values outside reference range are marked in bold. Bacterial species are indicated in italic font

m months, y years, n.d. not determined, *opson. opsonized*

A third patient (P3, family B), born to consanguineous Iraqi parents (Fig. 1a), presented at 7 months of age with hemolytic anemia in the context of a CMV infection which was successfully treated with corticosteroids/azathioprin and ganciclovir/valganciclovir as indicated by decreased CMV DNA levels. Direct Coombs test was positive and hemolytic anemia relapsed at the age of 27 months (Table 2) without detectable CMV DNA, prompting initiation of immunosuppressive treatment. Despite treatment, P3 developed transient thrombocytopenia (minimum 32 × 10^9^/L) which resolved spontaneously at the age of 3.5 years. Blood counts revealed reduced lymphocyte numbers (0.9–2.5 × 10^9^/L, Table 1) with low absolute numbers of T, B and NK cells, yet largely normal relative percentages of lymphocytes (Table 1). More in-depth immunophenotyping revealed slightly increased proportions of CD38^high^IgM^high^ transitional B cells (18.7%, reference 3.1–12.3% (ref. ^32^)) and CD38^high^IgM^-^ plasmablasts (7.7%, reference 0.4–4.0% (ref. ^32^))) but normal frequencies of CD19^+^CD27^+^IgD^−^ class-switched B cells (13.1%, reference 4.7–21.2% (ref. ^32^)), slightly reduced percentages of CD25^high^PD-1^low^FOXP3^+^ T_reg_ cells (Fig. S1f), and decreased mature CD56^dim^CD16^+^CD57^+^ NK cell population (Fig. S1g). Distribution of T-helper cell subsets in PBMCs, after accounting for age-related high numbers of naïve T cells, did not reveal abnormal skewing (Fig. S1h and i). Immunoglobulin levels were in the normal range (Table 1).

### Germline mutations in *DEF6* segregate in both families

**Fig. 2:**
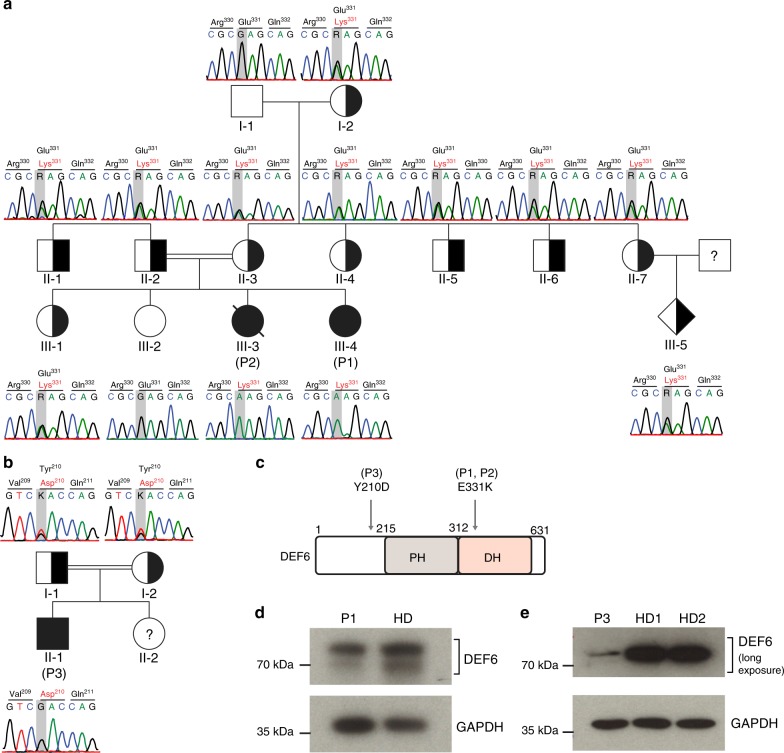
Distinct biallelic *DEF6* mutations segregate with disease. **a, b** Segregation pedigrees and chromatograms of the identified *DEF6* mutations in family A (a, variant c.G991A) and family B (b, variant c.T628G). All depicted individuals were validated by capillary sequencing. Unfilled – wild type; filled – homozygous mutation; half filled – heterozygous state.? – unknown genetic state (not sequenced). For information on the variants, see Supplementary Table S1. **c** Schematic of DEF6 protein domains indicating the identified mutations. PH – Pleckstrin homology domain; DH – Dbl homology domain. **d** DEF6^E331K^ mutant protein expression is partially reduced in feeder-expanded T cells of P1. **e** DEF6^Y210D^ mutant protein is barely detectable in T cells of P3 (long exposure is shown). Shorter-exposed immunoblots for (**e**) are shown in Supplementary Figure S2a. Immunoblots for (**d**) and (**e**) were cropped for visualization and are representative of two independent experiments. Source data of Fig. 2 including uncropped immunoblots are provided as a Supplementary Source Data file

Exome sequencing was performed for P1 and P3 to identify the underlying molecular disease etiologies, and confirmed by Sanger sequencing in respective family members. Among the segregating variants, *DEF6* was the single common gene affected in both pedigrees that segregated with the disease. Enlarged pedigrees were sequenced to confirm segregation of variants with disease (Fig. 2a, b). In family A, we identified a homozygous missense variant in *DEF6* (c.G991A, p.E331K) affecting the highly conserved PH-DH domain in both affected siblings P1 and P2 (Fig. 2a, c), while exome sequencing in family B identified a second, more N-terminal homozygous missense variant in *DEF6* (c.T628G, p.Y210D) in P3 (Fig. 2b, c). Genetic investigation was not performed for the newborn sister. Mutation Y210D affects a residue phosphorylated by ITK and necessary for interactions with the kinase^22^. This residue was previously shown to be phosphorylated by LCK as well and critical for induction of DEF6 activity^25^, however, these findings have not been corroborated by further studies. Both variants were predicted damaging by Polyphen-2, SIFT and CADD (Table S1). The identified *DEF6* mutations have not been reported in homozygous state in ExAC, gnomAD^33^ or TOPMed databases which are based on different population cohorts (Table S1), and heterozygotes were reported with minor allele frequencies below standard thresholds for rare diseases^34^ (Table S1). Probability of loss-of-function intolerance for DEF6 (pLI) was calculated as likely^33^ (Table S1). The mutated amino acids E331 and Y210 are conserved among vertebrates (Fig. S1j). Given the overlapping phenotypes, the identification of high-impact genetic variants in *DEF6* as the only gene found mutated in both pedigrees and segregating with the disease, and the assumed role of DEF6 in human immunity, we hypothesized that the *DEF6* variants were causative for the common disease phenotype. While amino acid exchange DEF6^E331K^ led to slight reduction in protein expression in P1-derived expanded T cells (Fig. 2d), DEF6^Y210D^ was barely detectable in feeder-expanded T cells of P3 (Fig. 2e and Fig. S2a). In summary, we identified three patients from two unrelated families presenting with features of systemic autoimmunity, bearing two distinct biallelic missense variants within the *DEF6* gene.

### *DEF6* mutations affect CTLA-4 cycling dynamics

As DEF6 is predominantly expressed in T cells (Fig. S2b), we focused on investigating T-cell phenotypes. While calcium flux was found unaltered in feeder-expanded patient cells upon TCR stimulation (Fig. S2c and d), ERK phosphorylation and AKT phosphorylation were partially reduced but not abolished compared to healthy donor (Fig. S2e). Intriguingly, proliferation of PBMCs or feeder-expanded T cells was not compromised (Fig. S2f and g). DEF6 is also expressed, to a lesser extent, in NK cells (Fig. S2b). No defect in NK-cell immunological synapse formation could be detected (Fig. S2h).

**Fig. 3:**
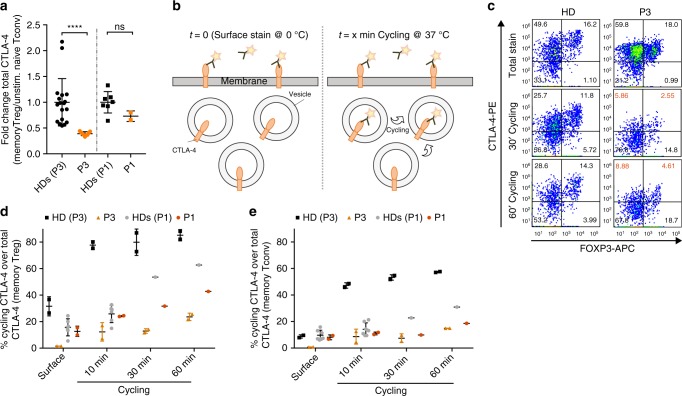
*DEF6* mutations affect CTLA-4 cycling. **a** Flow cytometric analysis of CTLA-4 expression in stimulated memory T_regs_ (CD4^+^CD45RA^−^FOXP3^+^CD25^+^), compared to unstimulated naïve T_conv_ as described in^37^. CTLA-4 expression in patients P1 and P3 was compared and normalized to respective healthy donor controls. Cells were stimulated for 16 h with anti-CD3/anti-CD28 antibody-coated beads. CTLA-4 expression after stimulation was reduced (P3) or unaltered (P1, n > 3). Data are overlaid with mean **±** SD. Statistics: ****p < 0.0001, ns: *p* = 0.067 (Welch’s *t* test). **b** Schematic representation of CTLA-4 cycling assay performed on purified CD4 T cells. **c**–**e** CD4 T cells of P1 and P3 show reduced CTLA-4 cycling, compared to HD. Representative FACS traces of memory T cells of P3 (**c**) and time-course quantifications of cycling traces of memory T_reg_ (**d**, CD4^+^CD45RA^−^FOXP3^+^) and memory T_conv_ cells (**e**, CD4^+^CD45RA^−^FOXP3^−^), normalized to respective total CTLA-4 expression of P1, P3 or HDs. Purified CD4 T cells were stimulated with anti-CD3/anti-CD28 antibody-coated beads for 16 h. Total stain was performed with standard intracellular staining. Cycling staining was performed by adding the labeled antibody before the cell harvest and incubation at 37 °C for the indicated times. Gating as in Fig. S3d. Representative of two independent blood shipments. Data are overlaid with mean ± SD. Source data of Fig. 3 are provided as a Supplementary Source Data file

CTLA-4, similar to DEF6, is predominantly expressed in T cells. Given the marked response of P1 to Abatacept (CTLA-4-Ig) treatment enabling clinical disease remission (Fig. 1c–e), we hypothesized that autoimmunity in DEF6 deficiency may be linked to aberrant CTLA-4 regulation. Expression of CTLA-4 is predominantly regulated by FOXP3 (ref. ^35^) and calcium-dependent NFAT activation^36^. We first analyzed CTLA-4 upregulation in stimulated memory-T_reg_ cells that most robustly express CTLA-4, and normalized the expression to unstimulated, naïve conventional T cells as previously described^37^. CD3/CD28 stimulation indeed showed significantly lower CTLA-4 expression in T_reg_ cells of P3, while CTLA-4 levels in P1 were non-significantly decreased (Fig. 3a and Fig. S3a). P1 and P3 both showed slightly reduced FOXP3 levels in T_regs_ (Fig. S1b and f). The checkpoint receptor CTLA-4 modulates T-cell responses through binding to and transendocytosis of the costimulatory molecules CD80/CD86 from APCs. While CD80/CD86 are degraded within T-cell lysosomes, CTLA-4 itself is recycled to the plasma membrane through the vesicular transport systems^8,10,11^. Defective CTLA-4 lysosomal sorting has been described previously to underlie autoimmunity in LRBA deficiency^11^. As DEF6 is a GEF for small GTPases, a protein class crucial for vesicular transports^38^, we focused on studying CTLA-4 trafficking processes in *DEF6*-mutated cells. To evaluate dynamic processes of CTLA-4 vesicular trafficking, we performed membrane cycling assays on primary T cells by comparing surface and cycled CTLA-4 normalized to total expressed CTLA-4, as outlined in schematic Fig. 3b. We observed reduced percentages of both surface and cycling CTLA-4 in all T-cell compartments of P3, including memory (CD45RA^−^) and naïve (CD45RA^+^), regulatory (FOXP3^+^) as well as conventional (FOXP3^−^) T cells, respectively (Fig. 3c–e and Fig. S3b–c, gating as in Fig. S3d). Analysis of P1 and corresponding healthy donors revealed reduced CTLA-4 cycling in the memory compartments (Fig. 3d, e). Defects in CTLA-4 cycling were observed despite normal activation as evidenced by CD25 upregulation (Fig. S3e). Comparing mean fluorescence intensities of cycled versus total CTLA-4 confirmed a relative reduction of cycling CTLA-4 in patient CD4 cells (Fig. S3f). We furthermore tested CTLA-4 re-cycling by only labeling for CTLA-4 that re-appeared on the cell surfaces after at least one cycle of antibody-tracked internalization, as outlined in schematic Fig. S3g. In line with our results, memory T_regs_ of P1 also showed a reduced appearance of re-cycled CTLA-4 at membrane surfaces, compared to healthy control (Fig. S3h and i). Altogether, CTLA-4 cycling processes were impaired in *DEF6*-mutated patient T cells.

### *DEF6* mutations affect CD80 ligand uptake by CTLA-4

**Fig. 4:**
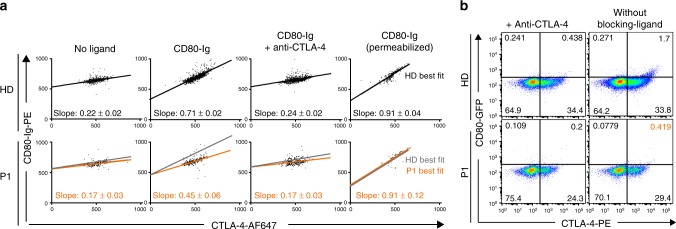
*DEF6* mutations affect CD80 ligand uptake by CTLA-4. **a** Ligand uptake assay of memory T_regs_ reveals reduced uptake of CD80-Ig in P1 cells (bottom, orange lines) compared to HD control (top and bottom, black/gray lines), as depicted by the reduced slope of the best-fit lines. Purified CD4 T cells were stimulated with anti-CD3/anti-CD28 antibody-coated beads (16 h). CD80-Ig and anti-CTLA-4 (where applicable) antibodies were present during stimulation. Anti-CTLA-4 blocked ligand uptake in both samples. For permeabilization control, CD80-Ig was added after fixation/permeabilization, for binding all available CTLA-4. Data from flow cytometry were extracted, visualized and analyzed with Prism. Slopes were calculated with linear regression. Gating as in Fig. S3k. Representative of two independent experiments. **b** Transendocytosis assay of CD4 T cells shows reduced CD80-GFP capture by P1 cells from CHO cells, indicated by reduced CTLA-4^+^CD80^+^ double-positive populations (orange numerical insert). Presence of anti-CTLA-4 blocked transendocytosis. Cells were stimulated for 16 h with anti-CD3 antibody. Co-stimulatory signal was provided by CD80-GFP expressing cells. Quantification as in Fig. S3j, gating as in Fig. S3l. Representative of two independent experiments. Source data of Fig. 4 are provided as a Supplementary Source Data file

We next investigated patient T cells for their ability to capture and trans-endocytose CTLA-4-ligands. We here investigated memory T_regs_, as previous work has shown that this cell population most robustly reveals defects in CTLA-4 ligand binding^37^. In accordance with our hypothesis that defective CTLA-4 cycling results in reduced surface availability of CTLA-4 and as a secondary consequence also in reduced relative ligand capture on the T-cell surface, we observed reduced uptake of CD80-Ig in P1 memory T_regs_ (Fig. 4a, gating as in Fig. S3k), as indicated by the reduced slope of the best fit line when compared to healthy controls (Fig. 4a, “CD80-Ig”). The observed differences are in line with previous reports on cells from patients with heterozygous *CTLA4* mutations showing dysfunctional ligand capture^37^. Presence of CTLA-4-blocking antibody abolished ligand uptake (Fig. 4a, “CD80-Ig + anti-CTLA-4”). Finally, the addition of ligand after cell permeabilization resulted in comparable slopes reflecting similar overall binding capabilities of the total expressed CTLA-4 (Fig. 4a, “CD80-Ig (permeabilized)”). Thus, our data demonstrate that CTLA-4 does not effectively reach surfaces in *DEF6*-mutated T cells, and as a result the surface-dependent function of CTLA-4 is disturbed. We also analyzed CTLA-4-dependent ligand transendocytosis from CD80-GFP expressing donor cells. In line with a reduced CTLA-4 surface abundance we found less CD80-GFP transendocytosed into CD4 T cells of P1 compared to healthy control (Fig. 4b and quantified for CD4 T cells in Fig. S3j, gating as in Fig. S3l). Presence of CTLA-4-directed antibody blocked transendocytosis (Fig. 4b and Fig. S3j). Altogether, due to impaired availability of the checkpoint protein CTLA-4 on T cell surfaces, the capturing and transendocytosis of ligands is consequently impaired.

### *DEF6* knockout phenocopies CTLA-4 cycling defects

**Fig. 5:**
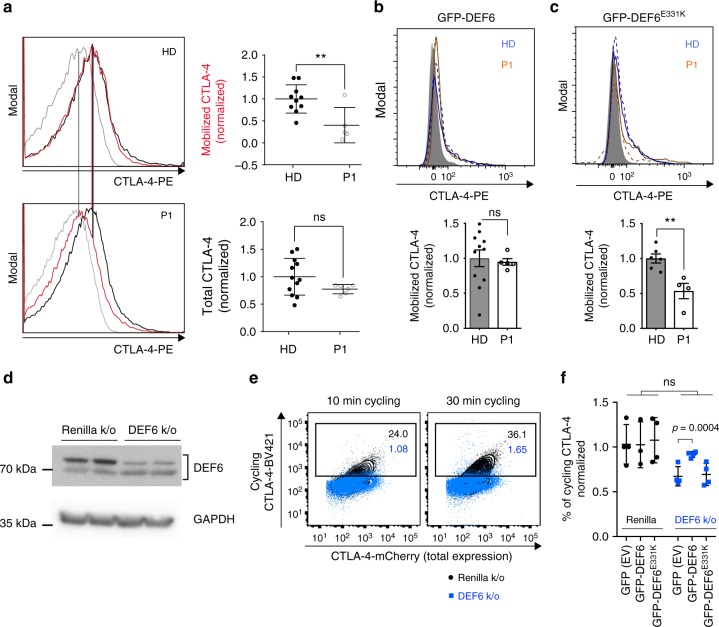
Defective CTLA-4 trafficking is rescuable and recapitulated by Jurkat *DEF6* knockouts. **a** Short-term mobilization of CTLA-4 is reduced in P1 T cells (bottom), compared to healthy control (HD, top). Cells were stimulated for 1 h with PMA/ionomycin. CTLA-4 antibody was present during stimulation to capture surface CTLA-4. Black – total CTLA-4, red – mobilized CTLA-4, gray – background surface stain. Statistics: **p = 0.0079, n.s. *p* = 0.1261 (Unpaired *t* test). Data are overlaid with mean ± SD. Two biological replicates. **b, c** Wild type but not mutant DEF6 rescues CTLA-4 mobilization defects in P1 cells. PBMCs were electroporated with either GFP-DEF6 (**b**) or with GFP-DEF6^E331K^ (**c**) and stimulated as in (**a**) (top: representative plots, blue: healthy control; orange: patient; dashed line: mobilized CTLA-4; straight line: total CTLA-4; bottom: quantification; Statistics: *p* = 0.7807 and *p* = 0.0043, unpaired t test). Data are overlaid with mean ± SD. Representative of two independent experiments. Gating as in Fig. S4a. **d** Immunoblot of CRISPR-mediated *DEF6* knockout and Renilla control, in Jurkat cells transduced with mCherry-CTLA-4. Images were cropped for visualization, an additional GAPDH blot is shown in the Source Data file. **e** Impaired CTLA-4 cycling in *DEF6* knockout cells. Clones expressing mCherry-CTLA-4 were stimulated overnight with anti-CD3, and incubated with anti-CTLA-4 for 10 or 30 min, respectively. *DEF6* knockout impairs CTLA-4 cycling (blue), compared to Renilla (black). Numerical inserts represent percentages of cycled CTLA-4. Gating as in Fig. S4b. Representative of three independent experiments. **f** Overexpression of wildtype but not mutant DEF6 partly rescues defective CTLA-4 cycling in Jurkat *DEF6* knockout cells. Cells were electroporated with constructs before stimulation and cycling analysis as in (**e**). Data were normalized to CTLA-4 cycling of GFP (EV) transfected Renilla k/o cells. Representative of two independent experiments. Data are overlaid with mean ± SD. Statistics: Multiple *t* test, FDR 1%. Gating as in Fig. S4c, only transfected (GFP positive) cells were used for cycling analysis, expression levels of GFP are shown in Fig. S4d. Source data of Fig. 5 including uncropped immunoblots are provided as a Supplementary Source Data file

To evaluate the causality of mutated DEF6 for aberrant regulation of CTLA-4 trafficking, we utilized several models. We performed CTLA-4 mobilization assays on CD4 T cells of P1 and a healthy donor to monitor CTLA-4 on cell surfaces after short stimulation, which effectively mobilizes CTLA-4 from internal stores. While total CTLA-4 expression was unaffected, mobilized CTLA-4 was reduced in patient T cells compared to HD (Fig. 5a, gating as in Fig. S4a). In a similar setup, we electroporated healthy control or DEF6^E331K^-mutated PBMCs with wild type DEF6-GFP (Fig. 5b) or mutated DEF6^E331K^-GFP (Fig. 5c). The observed mobilization defect of CTLA-4 in DEF6^E331K^-mutated CD4 T cells was reversed by wild-type DEF6 but not by DEF6^E331K^ (Fig. 5b, c, gating as in Fig. S4a). To further prove causality, we generated CRISPR-mediated knockout clones of DEF6 and Renilla control in CTLA-4-mCherry transduced Jurkat cells (Fig. 5d). Pronounced reduction of DEF6 expression resulted in defective CTLA-4 cycling, confirming the role of DEF6 in regulating CTLA-4 trafficking (Fig. 5e, gating as in Fig. S4b). These defects could be partly reconstituted by electroporating wildtype but not mutant DEF6 (Fig. 5f, gating as in Fig. S4c), though slightly more mutant protein was expressed (Fig. S4d). *DEF6* knockout cells furthermore showed reduced suppression tendency against CD4 target cells in presence of unlabeled PBMCs as APC source (Fig. S4e, gating as in Fig. S4f).

Our collective data on primary T cells reconstituted with wildtype DEF6 and on Jurkat knockout cells demonstrate that the decreased CTLA-4 availability in *DEF6*-mutated patient cells is caused by defective intracellular trafficking processes.

### DEF6 mutations affect RAB11 interactions

**Fig. 6:**
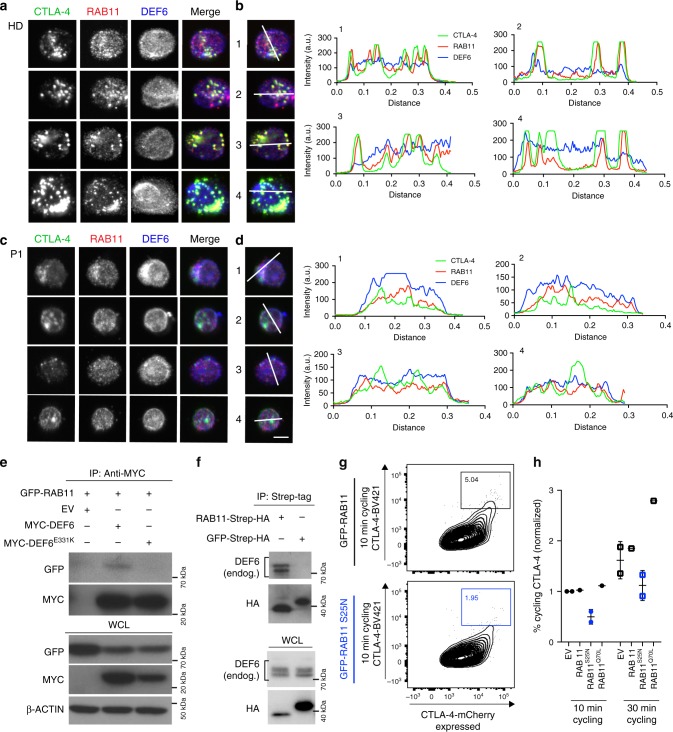
DEF6 mutations affect RAB11 interactions. **a** Representative images of endogenous CTLA-4, and RAB11 and DEF6 in TCR-stimulated healthy control (HD) PBMCs showing RAB11-CTLA-4 co-localization. **b** Line scans of images in (**a**) reveal high overlap of RAB11 and CTLA-4 signal in activated HD-PBMCs. Scale bar – 5 µm. **c** Representative images of endogenous CTLA-4, RAB11 and DEF6 in activated PBMCs of P1 reveal loss of RAB11-CTLA-4 co-localization in P1. **d** Line scans show reduced overlap of RAB11 and CTLA-4 signal in P1. Scale bar – 5 µm. (for a-d, representative images of 30–40 analyzed cells; cells were considered T cells through expression of CTLA-4 after TCR cross-linking). Quantification as in Fig. S5a. **e** MYC-tagged wildtype DEF6 co-immunoprecipitates with GFP-RAB11 from transfected HEK293T cells, revealing a hitherto unrecognized interaction of the GEF protein DEF6 with the small GTPase RAB11. Presence of mutation E331K abrogated this interaction. Samples were balanced on immunoprecipitated GFP-RAB11 fractions and blotted for interacting DEF6. Representative of three independent experiments. **f** Endogenous DEF6 co-immunoprecipitates from Jurkat lysates with overexpressed RAB11-Strep-HA, compared to GFP-Strep-HA control. Samples were balanced on immunoprecipitated HA-tag fractions. Western blots (**e**, **f**) were cropped for visualization. **g**, **h** Overexpressing inactive RAB11^S25N^ in Jurkat-mCherry-CTLA-4 cells mimics DEF6-deficient defects in CTLA-4 cycling. Cells were electroporated with inactive RAB11^S25N^, constitutively active RAB11^Q70L^, wildtype RAB11 or empty vector (EV), stimulated with OKT3 and analyzed for cycling CTLA-4 after 10 min and 30 min, respectively. Cycling was reduced in RAB11^S25N^ expressing cells (blue, blue numerical insert, g), while RAB11^Q70L^ enhanced cycling at 30 min (**h**). Data are overlaid with mean ± SD. Representative of two individual experiments. Source data of Fig. 6 including uncropped immunoblots are provided as a Supplementary Source Data file

Given our finding that the guanine nucleotide exchange factor DEF6 regulates CTLA-4 cycling processes, we hypothesized that DEF6 might regulate the small GTPase RAB11, a central protein for recycling endosomes that has been shown to co-localize to CTLA-4^+^ vesicles^8,11^. We first assessed the localization of endogenous DEF6, RAB11 and CTLA-4 in activated patient-derived and control PBMCs. In line with previous studies^8^, we observed prominent co-localization of CTLA-4 with RAB11 in the healthy control cells (Fig. 6a, b). In sharp contrast, RAB11/CTLA-4 co-localization was largely reduced in TCR/CD28-stimulated DEF6^E331K^-mutated cells of P1 (Fig. 6c). Line scans through CTLA-4^+^ vesicles confirmed the lack of co-localization with RAB11 in DEF6^E331K^-mutated cells (Fig. 6d). To quantify RAB11 and CTLA-4 co-localization, defined regions of interest with high CTLA-4 expression and RAB11-positive vesicles were selected and analyzed for overlap coefficients. Quantitative analyses revealed a significant reduction of co-localization for P1 and P3 compared to respective healthy control cells (Fig. S5a-c), suggesting a negative impact of mutated/reduced DEF6 on CTLA-4^+^RAB11^+^ recycling vesicles. Of note, RAB11 was expressed at similar levels in P1 and P3 as in healthy controls (Fig. S5d). To validate changes in interaction of wildtype or mutated DEF6 with RAB11, we performed co-immunoprecipitation analyses in co-transfected HEK293T cells. While wildtype DEF6 co-immunoprecipitated with RAB11 (Fig. 6e), this interaction was reduced in the DEF6^E331K^-expressing cells (Fig. 6e). These results suggest a possible GEF activity of wildtype DEF6 for the small GTPase RAB11, and could hence play a causative role in reduced recycling of CTLA-4 in patient cells. Consistent with the fact that GEF proteins for small GTPases interact preferentially with the dominant negative, GDP-bound form of their target proteins^39^, we found that DEF6 interacted strongly with dominant-negative (GDP-locked) RAB11^S25N^, but weakly with constitutively-active (GTP-bound) RAB11^Q70L^ in HEK293T cells (Fig. S5e). In contrast, DEF6^E331K^ did not show relevant co-immunoprecipitation with wild-type, GDP- or GTP-locked RAB11 (Fig. S5f, long exposure shown). In Jurkat cells, endogenous DEF6 co-immunoprecipitated with Strep-HA-tagged RAB11 but not with the Strep-HA-GFP-expressing control (Fig. 6f), confirming a physical interaction in a T-cell model. The second identified mutation DEF6^Y210D^ was barely expressed in primary T cells of P3 (Fig. 2e), probably due to rapid degradation. We overexpressed GFP-tagged wildtype or Y210D-mutated DEF6 in Jurkat cells in presence or absence of proteasomal inhibitor MG132 and tracked GFP signals over time. Our data confirm loss of DEF6^Y210D^ two days after transfection which could be fully reverted by adding MG132 (Fig. S5g). Lastly, kinetic studies on purified proteins suggest that the PH-DH domain of DEF6 has GEF activity toward the small GTPase RAB11, while mutated PH-DH^E331K^ is inactive (Fig. S5h).

To confirm the link between RAB11 and CTLA-4 trafficking, we overexpressed inactive RAB11^S25N^ and constitutively active RAB11^Q70L^ in Jurkat-mCherry-CTLA-4 cells, and analyzed CTLA-4 cycling by counterstaining the cycling protein with BV421-coupled antibody. As shown in Fig. 6g, h (gating as in Fig. S5i), when compared to overexpressing wildtype RAB11 or empty vector, inactive RAB11^S25N^ indeed blocked CTLA-4 cycling while presence of active RAB11^Q70L^ contrarily enhanced this process at longer time points.

In sum, our data reveal a previously unknown physical interaction and GEF activity of DEF6 toward the small GTPase RAB11, a recognized component of CTLA-4^+^ recycling endosomes^8^. Consistently, DEF6-mutated cells lacked this interaction and showed reduced RAB11^+^CTLA-4^+^ double-positive vesicles, suggesting a direct link to the observed defect in CTLA-4 trafficking dynamics through RAB11 as demonstrated by inactive RAB11^S25N^ compromising CTLA-4 cycling.

## Discussion

The role of DEF6 in murine autoimmunity models has been controversial as the development of autoimmunity appears to depend on their genetic background^26,27,30,31^. In humans, the intronic *DEF6* SNP rs10807150 which alters gene expression, is associated with the onset of systemic lupus erythematosus^40^. Here, we describe two unrelated families with three patients harboring two distinct biallelic missense mutations in *DEF6*. The patients present with immunodeficiency and systemic autoimmunity, thus indicating a critical role for DEF6 in preventing autoimmunity in humans. We uncover a role for DEF6 in regulating abundance and recycling of the T-cell checkpoint protein CTLA-4, as the functional cause of the observed autoimmune manifestations in *DEF6*-mutated patients. We base our conclusions on the following observations and in line with previously outlined criteria:^41^ (i) we identified different biallelic mutations in *DEF6* as the single common gene affected and segregating perfectly with the disease in two unrelated families; (ii) DEF6 has been previously shown to have a role in the immune system although its precise role in human immunity had not been determined; (iii) we identified a CTLA-4 trafficking defect amenable to rescue upon reconstitution of patient T cells with wildtype DEF6, explaining the predominant clinical presentation of autoimmunity; (iv) CRISPR-based *DEF6* knockout in Jurkat cells recapitulates defective CTLA-4 cycling and could be reverted by reconstitution with the wildtype protein; (v) we provide a functional explanation involving compromised RAB11-DEF6 interaction affecting RAB11-dependent CTLA-4 shuttling; (vi) lastly, the successfully commenced CTLA-4-Ig therapy in P1 led to remission of symptoms.

CTLA-4 is a critical molecule in human immune homeostasis. A reduction of CTLA-4 levels by 50% as observed in CTLA-4 haploinsufficiency results in severe autoimmunity^13,14^, while notably patients with biallelic loss-of-function germline mutations in *CTLA4* have not been described and are potentially lethal. *Ctla4*^*−/−*^ mice are viable, although they develop fatal autoimmunity early in life whereas their *Ctla4*^*+/-*^ littermates are healthy^42,43^. These studies suggest that humans appear to have a narrower window of tolerance regarding CTLA-4 critical abundance for the onset of disease. This assumption is further supported by genome-wide association studies which have identified SNPs affecting the relative cell surface expression of CTLA-4 associated with human autoimmune disease^44^. Reduction of available CTLA-4 by enhanced lysosomal degradation is also the cause for severe autoimmunity in LRBA deficiency^11^. Again, in contrast to the human phenotype, *Lrba*^*−/−*^ mice do not develop overt autoimmunity^45–47^, have a normal lifespan and also intriguingly exhibit an increased acceptance of allogeneic bone marrow grafts^45^. Thus, *Def6*^*−/−*^, *Ctla4*^*−/+*^, and *Lrba*^*−/−*^ mice display inconsistent autoimmune manifestations or lack such. Our discovery of a mechanistic link between *DEF6* mutations and CTLA-4 functional integrity offers insights to autoimmunity in humans. Clinical and immunological phenotypes in *DEF6*-mutated patients include T-cell lymphopenia, low class-switched B cells, hepatosplenomegaly, autoimmune hemolytic anemia and bowel inflammation, all of which are reminiscent of CTLA-4 haploinsufficiency and LRBA deficiency^13–18,48–50^. In accordance with previous reports on genetically determined autoimmune diseases through compromised CTLA-4, clinical manifestations vary between patients due to the lowered thresholds of inhibitory T cell function, rather than through specific triggers^51^. Still, they do represent the same disease. As for other newly described disease entities, larger patient cohorts in future studies will help to unravel the full phenotypic spectrum of disease due to functional DEF6 deficiency. It is impossible to dissect whether the strong immunosuppressive treatment in P1 may have contributed to the more pronounced B-cell deficiency including borderline-low frequencies of class-switched memory B cells and impaired vaccination titer generation, and also the persistent susceptibility to infections which has been described previously in individuals treated with abatacept^52^. To date, P3 has exhibited less pronounced autoimmune manifestations. This could be due to a distinct mutation with distinct cellular effect, or possibly a different genetic or epigenetic background. Given the reduced CTLA-4 expression in P3 (Fig. 3a), it is possible that other autoimmune manifestations may present with time. Interestingly, in contrast to CTLA-4 and LRBA-mutated patients, DEF6-mutated patients do not show an obvious activation/exhaustion phenotype in peripheral blood T cells. This might be due to the fact that DEF6 is also involved in T-cell signaling. Def6^−/−^ mice, for example, exhibit a reduced clonal expansion of CD8^+^ T cells^29^. The interplay of DEF6 in T-cell signaling and regulation of CTLA-4 might result in a normal status of T cells derived from the blood, but increased activation in situ, where antigen is presented in higher concentrations as suggested by the massive T-cell infiltration in peripheral tissues (Fig. 1b). The homozygous frameshift mutation in *SKIV2L* that was additionally identified in P1 and the deceased sister P2 in family A (Table S1), could represent a disease-modifying factor potentially affecting cardiac function and/or bowel inflammation^53^, but does not explain the autoimmune presentation observed in *DEF6*-mutated individuals from both families and our identified link to aberrant CTLA-4 shuttling. We proved causality by reconstitution of the CTLA-4 cycling defect in patient-derived cells through ectopic expression of wildtype DEF6, and a similar reconstitution of *DEF6*-knockout Jurkat models could revert the observed CTLA-4 cycling defect. Finally, the response of P1 to CTLA-4 replacement therapy suggests a T cell-mediated disease.

The co-localization, co-immunoprecipitation and overexpression data confirm that DEF6 regulates CTLA-4 vesicular trafficking via the small GTPase RAB11. RAB11 has previously been located at recycling vesicles containing CTLA-4^8^. We identify a cellular regulation pathway of CTLA-4, which may involve direct activation of RAB11 by DEF6, a GEF protein that functions downstream of TCR engagement. RAB11 is a broadly expressed small GTPase and its deletion in a murine knockout model was found embryonically lethal^54^. It is also considered a crucial component of the so-called exocyst, which regulates late-endosomal trafficking^55^. However, it is largely unknown which GEF proteins activate RAB11 to promote its multiple actions, and future studies are required to address this question. Our data reveal that RAB11 interacts with DEF6, and that DEF6 acts as GEF protein for RAB11 as suggested by a preferential interaction of DEF6 with the GDP-locked small GTPase, and further evidenced by kinetic GEF activity studies on purified protein domains. The phenocopy of defective CTLA-4 cycling by overexpressing inactive RAB11^S25N^ in Jurkat cells further supports this theory. In patient-derived *DEF6*-mutant T cells, RAB11^+^ recycling endosomes do not co-localize with CTLA-4^+^ vesicles, suggesting that DEF6 promotes RAB11-mediated recycling of CTLA-4.

In conclusion, our work identifies a role for DEF6 in regulating CTLA-4 availability and trafficking to prevent autoimmunity, in line with CTLA-4 functioning both as immune rheostat and defining thresholds of immune activation for anti-cancer immunity^11,13,14,56^. The work presented herein underlines the power of identifying genetic causes for immune diseases as a way to uncover immune regulatory pathways^2^. Given the identified role of DEF6 in tuning the immune checkpoint protein CTLA-4, future studies should address whether DEF6 and related proteins are amenable to manipulation for targeted therapeutic intervention in immune-mediated disorders or potentially also anti-cancer immunotherapeutic approaches.

## Methods

### Patients and ethics

Patient studies have been approved by the ethics committee at the Medical University of Vienna (MedUni Vienna), Austria (study number EK499/2011), and the Regional Ethical Review Board in Stockholm (study number 2013/1723–31/4). Patients P1 and P2 were evaluated, followed up and treated at the Children’s hospital of the MedUni Vienna. P3 was evaluated, followed up and treated at Astrid Lindgren’s Childrens Hospital in Stockholm. Biological material of patients and healthy donors (HD) was obtained on informed consent in accordance with the Declaration of Helsinki. Blood drawings were taken by venipuncture. Healthy-donor shipping controls which underwent the same handling and storage conditions were included in all experiments where blood was not directly assessed.

### Further clinical information on the patients

Patient 1 (P1, index patient of family A) presented oligohydramnion in prenatal ultrasounds and intrauterine growth retardation. A Caesarean section was performed at 38 weeks of gestation due to rupture of the membrane and pathological dopplersonographic measurements. Weight and length at birth were below third percentile (1435 g; 39 cm; head circumference 25.5 cm). No abnormalities were observed during the perinatal period, apart from mild respiratory distress syndrome. P1 presented hypertelorism, inward mamillae, growth retardation (below third percentile) and abnormal fatty tissue distribution. At 23 days of life severe watery diarrhea was observed, associated with vomiting and electrolytes imbalances (hypernatremia and hyperchloremic acidosis; Na: 159 mmol l^−1^, pH 7.1; base excess: −18) and massive increase of inflammation markers (C reactive protein concentration: >20 mg dl^−1^). Total parenteral nutrition was initiated with no obvious improvements of diarrhea. Hydrolyzed formula also did not improve P1’s health status. Viral, bacterial, parasitic or allergic causes of the diarrhea were excluded upon repeated testing. Massive bowel inflammation was suggested by increased stool calprotectin (Fig. 1c). Endoscopy revealed atrophy of gastric mucosa with numerous apoptotic cells. Complete villous atrophy was also observed in the small intestine. Microvillus inclusion disease and an underlying metabolic disease were excluded. The duodenum showed massive infiltration of eosinophils and T cells (Fig. S1a). Plasma and goblet cell numbers were reduced. Colonic mucosa showed normal appearance. Subsequently, P1 developed perianal dermatitis (Fig. 1e). Topical treatment had no effect on the dermatitis and deep, indurated, painful ulcers evolved. Enteral feeding was offered, but led to massive vomiting. Thus, therapy with corticosteroids (prednisone 1 mg kg^−1 ^day^−1^) was initiated which showed slight improvement of disease status. Intravenous cyclosporine was added to the therapy. Enteral feeding was gradually increased through a jejunostomy. Vomiting ceased and stool consistency improved substantially. Parenteral nutrition could be withdrawn. With subsequent reduction of cyclosporine, watery diarrhea recurred leading to a loss of about 1.5 kg within two weeks. At the age of 2 months P1 presented hepatomegaly and laboratory liver abnormalities (elevated γ-glutamyl transferase (450 U l^−1^) and liver enzymes (AST/ALT: 100 U l^−1^)), which were treatable with ursodeoxycholic acid. Echocardiography imaging of P1 revealed a dilated cardiomyopathy with an atrial septal defect (ASD). Treatment with phosphodiesterase inhibitor and acetylsalicylic acid was initiated. Due to suspected vasculitis the patient further received intravenous immunoglobulins. P1 developed a biventricular hypertrophy, with an ASD. Treatment with enalapril, atenolol, spironolactone, and furosemide resulted in improved ventricular function, however, biventricular hypertrophy persisted and arterial hypertension developed. Due to reduced immunoglobulin levels after birth (Table 1), the patient was supplemented with intravenous immunoglobulins (IVIG) for two months in which IgG/IgM/IgA levels were closely monitored. IVIG administration was paused until the age of 10 months when IVIG supplementation was reinitiated due to reduced specific immunoglobulin titers after vaccination with various agents (polio, diphtheria, tetanus) (Table 2).

Patient 2 (P2, deceased sister, family A) was born at 35 weeks of gestation due to premature rupture of the membrane. She showed intrauterine growth retardation (weight at birth: 1260 g; length at birth: 38 cm (both: below third percentile for age); and head circumference at birth: 29.5 cm (at third percentile)) without catch-up postnatally (weight at 2 months of age: 1696 g; length at 2 months of age: 40 cm (both: below third percentile for age). She presented with a cleft palate. Enteral feeding was difficult due to clinical signs that were interpreted as necrotizing enterocolitis. Neither diet with extensively hydrolyzed formula nor with elemental formula improved her clinical condition, necessitating parenteral nutrition. Colonoscopy at the age of 138 days revealed rectal ulcers and remarkably few plasma cells and increased numbers of apoptotic cells in the descending and transverse colon. Rectal fissures and ulcers were detected. Mucosal membrane showed normal conditions. Besides the gastrointestinal symptoms, the patient was found to have an atrioventricular septum defect (AVSD) which was treated with pulmonary artery banding and VSD patch closure. She developed increasing heart insufficiency, pulmonary hypertension and a third-grade atrioventricular (AV) block requiring pacemaker treatment. Her liver presented severe hepatomegaly and signs of progressing cholestasis, siderosis, steatosis and hypertriglyceridemia (430 mg dl^−1^). Furthermore, she developed a metabolic acidosis and hypokalemia. Screening for an underlying metabolic disorder was negative. Liver failure was reported as P2 presented massive jaundice, anasarca and ascites. Histology of the liver revealed autolysis. Clinical signs and symptoms suggestive of an undefined immunodeficiency appeared as P2 presented with recurrent infections and sepsis (Table 2). Autopsy after death revealed pulmonary artery and biventricular dilation as well as right ventricular hypertrophy. She showed pleural effusion and congestion.

Patient 3 (family B) was born uneventfully and healthy except for a verrucous nevus. His presentation with CMV initiated the treatment with corticosteroids as well as ganciclovir/valganciclovir. EBV and HIV serology were negative. As CMV DNA levels decreased, valganciclovir treatment was stopped after 1 month of therapy. Azathioprine treatment started after six weeks, during tapering of steroids. The steroids were stopped after 4 months of treatment, with azathioprine continued for an additional 3 months. During the relapse of hemolytic anemia P3 was put on immunosuppressive treatment again with corticosteroids and azathioprine until the age of 3.5 years. During treatment he developed transient thrombocytopenia (minimum 32 × 10^9^ L^−1^) at the age of 2 years and 4 months. Thereafter, he has remained with a growth curve without remarks (5.5 years currently). Blood values have normalized except for lymphocyte numbers being repeatedly low (0.9–2.5 × 10^9^ L^−1^, Table 1). The patient has not displayed any gastrointestinal symptoms but was prescribed oral antibiotics since, and no cardiac anomalies were detected.

### Genetic analysis

DNA of P1 and P3 was extracted from whole blood with Genomic DNA Purification kits (Promega). DNA of P2 was extracted after death from stored histology slides. DNA of relatives was extracted either as described for P1 or from saliva samples with the QIAamp® DNA mini kit. For P1 (family A), homozygous intervals were determined applying Affymetrix® SNP-based homozygosity mapping and used as a filter for detected variants. Whole exome sequencing was performed on genomic DNA of P1 and analyzed for novel non-sense, missense and frameshift variants, as follows: After library prep with the Illumina True Seq and Exon Enrichment kit, the sample was multiplexed and loaded onto two lanes of one flow cell. DNA was sequenced on an Illumina HiSeq2000 Sequencer by paired-end sequencing. After variant calling, demultiplexing and alignment of the 227,048,916 reads, 97.8% of reads could be uniquely mapped. Average target coverage was 140×. VCF.Filter software^57^ was used for filtering for missense, nonsense, splice-site and frameshift variants in the whole exome sequencing data. The obtained list was filtered to exclude variants with a minor allele frequency (MAF) > 0.01 in 1000 Genomes, dbSNP and gnomAD, and an internal cohort database was used to further exclude recurrent variants. Filtered candidate variants were analyzed and investigated in the ExAC and gnomAD browsers for loss-of-function intolerance and predicted-to-observed mutation rates^33^. For segregation analysis of candidate variants, DNA of family members (14 individuals, Fig. 2a) was investigated by Sanger sequencing, leaving three candidate genes with variants in family A (Supplementary Table S1). For family B, whole-exome enrichment and sequencing of P3 and the healthy mother were performed applying a Agilent SureSelect v5 51 Mb kit and Illumina HiSeq2000 sequencer. Reads were aligned to human genome GRCh37 with BWA/0.7.4 (ref. ^58^). Variant calling and annotation were performed with haplotype caller from the Genome Analysis Toolkit (GATK) (v.3)^59^ and variant effector predictor (VEP) (version 75)^60^, respectively. Variant filtering was performed using GEMINI (v0.11.0a)^61^. Validation of variants was done by standard capillary sequencing, family members (parents) were similarly sequenced for presence of identified variants (Fig. 2b). *DEF6* remained as the single gene affected and segregating in both pedigrees (Supplementary Table S1). All next generation sequencing data are deposited at the EGA database with restricted access (see the *Data Availability* section). Analyses of conservation and mutational impact were done with the prediction tools Polyphen-2^62^ and SIFT^63^, and CADD scores^64^ were calculated for segregating genes.

### Flow cytometry

For family A, immunophenotyping was performed on a BD LSRFortessa^TM^ or BD FACSCanto^TM^II. Peripheral blood mononuclear cells (PBMCs) were isolated from patients or HD blood with a Ficoll gradient and were either used fresh or cryo-preserved in liquid nitrogen. Staining of surface antigens was performed after blocking in FBS-containing medium for 30 min at 4 °C, intracellular antigens were stained applying the fixation/permeabilization kit for intracellular antigens or transcription factors (Affymetrix, eBioscience). All analyses were performed using FlowJo X (TreeStar Inc.) and Prism 7.0 (GraphPad Software). Magnetic microbeads-based sorting of total CD4 T cells was done by negative depletion applying the EasySep human CD4^+^ T cell enrichment kit, stem cell technologies^TM^. The following antibodies were used for flow cytometry: From Beckman Coulter: CD16-FITC (clone 3G8), CD19-PECy7 (J3–119), CD3-FITC (UCHT1), CD3-PC5.5 (UCHT1), CD4-PE (13B8.2), CD4-PECy7 (SFCI12T4D11), CD45RA (ALB11), CD45RO (UCHL1), CD56-PE (N901), CD56-PECy5 (N901), CD8-PECy7 (SFCI21), TCRvα24-PC-7 (C15), TCRvβ11-FITC (C21); from eBisocience, Affymetrix: CD152-PE (14D3), CD19-PerCPCy5.5 (HIB19), CD3-APC (SK7), CD3-APC (UCHT1), CD4-eFluor450 (RPA-T4), CD4-PerCPCy5.5 (RPA-T4), CD45RA-PerCP-Cy5.5 (HI100), CD69-APC (FN50), FOXP3-FITC (PCH101), FOXP3-eFluor450 (236A/E7), FOXP3-APC (236A/E7) from BD Biosciences: CD152-PE (BN13), CD16-PECy7 (3G8), CD19-PECy7 (SJ25C1), CD25-PE (M-A251), CD25-BV605 (2A3), CD27-PE (M-T271), CD27-PECy7 (M-T271), CD27-V450 (M-T271), CD3-APC-H7 (SK7), CD4-APC (RPA-T4), CD4-AF700 (RPA-T4), CD4-BV421 (RPA-T4), CD4-BV605 (RPA-T4), CD45RA-AF700 (HI100), CD45RO-FITC (UCHL1), CD56-AF700 (B159), CD56-V450 (B159), CD69-APC (L78), CD69-PECy7 (L78), CD69-PECy7 (FN50), CD8-V450 (RPA-T8), CD8-V500 (RPA-T8), CD8-FITC (HIT8a), Igκ-PE (G20–193), IgD-APC-H7 (IA6–2), IgD-FITC (IA6–2), TCRαβ-FITC (WT31), TCRαβ-PE (T10B9.1A-31), TCRγδ-APC (B1), TCRγδ-PE (11F2).

For immunophenotyping of P3, PBMCs from P3 and healthy controls were obtained by Ficoll gradient isolation. Extracellular antigens were stained at room temperature for 20 min in FACS Buffer (PBS + 2% FCS + 4 mM EDTA) supplemented with directly conjugated antibodies, fixed with 1.6 % formaldehyde, permeabilized and stained intracellularly for 20 min at room temperature in BD Perm/Wash Buffer (BD Biosciences) supplemented with directly conjugated antibodies. Alternatively, fixation and intracellular staining was performed with the FoxP3/Transcription Factor staining buffer set (eBiosciences) according to protocol. Cells were analyzed on a BD LSRFortessa and all analysis was perfomed using FlowJo software (TreeStar). To assess absolute numbers of immune cell subsets, Trucount assays were performed (BD Biosciences). The following antibodies were used for P3: From Beckman-Coulter: TCRgd-FITC (clone IMMU510) CD27-PECy5.5 (1A4CD27) CD27-ECD (1A4CD27), TCRVα24-FITC (C15), TCRVβ11-APC (C21), CD56-PECy5.5 (N901), NKG2A-PECy7 (Z199), CD27-PECy5.5 (1A4CD27); from BD Biosciences: CD45-V500 (HI30), IgD-BV711 (IA6–2), CD38 (MHN4–2), CD16-PECF594 (3G8), CD56-PECy7 (NCAM16.2), CXCR5-AF488 (RF8B2), CD8-APCCy7 (SK1), FOXP3-V450 (259D/C7), CCR4-PE (1G1), CD69-BUV395 (FN50), CCR6-BUV737 (11A9), CD16-APCCy7 (3G8), CD16-V500 (3G8), CD19-V500 (HIB19 BD), IgM-APC (G20–127), CD16-AF700 (3G8), CD38-BV785 (HIT2), CXCR4-PE (12G5), CD25-PECy7 (M-A251), streptavidin-FITC (554060), CD19-APCCy7 (SJ25-C1), CD57-BV605 (NK-1); from Biolegend: CD45RA-A700 (HI100), CD8-BV570 (RPA-T8), CD3-BV605 (OKT3), PD-1-APC (EH12.2.H7), TCRγδ-BV510 (B1), CD3-BV711 (OKT3), CD45RA-BV785 (HI100), CXCR3-PE-Dazzle (G025H7), CD57-Pacific Blue (HCD57), CD19-FITC (HIB18), IgD-APCCy7 (IA6–2), CD25-APC (BC96), CD45RO-A700 (UCHL1), CCR7-BV421 (G043H7), CD8-BV711 (RPA-T8); from Invitrogen: CD4-Qdot605 (S3.5); from Exbio Antibodies: CD21-Pacific Blue (LT21); from Miltenyi Biotec: NKG2C-biotin (REA205).

### Cell culture and stimulation conditions

PBMCs and –derived cells as well as Jurkat E6.1 cells were maintained in RPMI-1640 medium supplemented with 10% of heat-inactivated FCS (Life Technologies, Gibco), 50 U ml^−1^ penicillin, 50 mg ml^−1^ streptomycin and HEPES (all from Gibco) at 37 °C in a humidified atmosphere with 5% CO_2_. For CD4 T-cell isolation, Affymetrix eBioscience MagniSort negative selection was used. T cells were either stimulated with CD3/CD28 antibody-bearing dynabeads (Gibco) at a 1:1 or 1:2 bead to cell ratio, or with anti-CD3 (OKT3) and anti-CD28 (CD28.2) soluble antibodies (both from eBioscience). T cells were expanded by co-culturing with gamma-irradiated feeder cells^65^ with PMA/ionomycin stimulation. CHO cells were cultured in DMEM medium (Gibco), supplemented as above.

### Immunoblots

Cells were lysed in IP buffer (20 mM Tris (pH 7.5), 150 mM NaCl, 2 mM EDTA, 1% TritonX-100, phenylmethylsulfonylfluoride (PMSF, 100 mM), complete protease inhibitor cocktail (PIC)) or RIPA buffer supplemented with PMSF and PIC, and analyzed by Western blot with primary antibodies against: DEF6 (H00050619-B01; Abnova), GAPDH (sc-365062; Santa Cruz Biotechnology), RAB11 (700184, Invitrogen), HSP90 (F-8, Santa Cruz). Uncropped scans and (where applicable) additional exposures of all immunoblots are shown in a separate *Source Data* file, with molecular weight markers indicated.

### Phosphoblotting

Feeder-expanded T cells were starved for 4 h in PBS/0.5% human serum. Samples were placed on ice and PHA stimulation mix was added still on ice. Samples were placed on a 37 °C thermoshaker and at the indicated time points, ice-cold PBS was added and respective samples were placed on ice immediately. Cells were then lysed in RIPA buffer as above, and resolved by SDS-PAGE Western blot. The following primary antibodies were used: HSP90 (F-8, Santa Cruz), AKT (40D4, Cell Signaling), phospho-AKT (Ser473, D94, Cell Signaling), ERK1/2 (137F5, Cell Signaling), phospho-ERK1/2 (Thr202/Tyr204, Cell Signaling).

### mRNA expression

Extraction of RNA was performed using RNeasy kit (Qiagen), first-strand complementary DNA synthesis was done using Expand Reverse Transcriptase (Roche) using both oligo-dT and random hexamer primers. Intron-spanning primers were used for gene expression analysis: *DEF6*-forward: 5′- CATCTCGGAAGTGTTCCTCC-3′, *DEF6*-reverse: 5′-CAAGTCCATCTGGTACGCCT-3′, *ACTB*-forward: 5′-GTTGTCGACGACGAGCG-3′, *ACTB*-reverse: 5′-GCACAGAGCCTCGCCTT-3′.

### Calcium flux

To assess calcium influx in P1, feeder-expanded T cells were harvested, washed with PBS and loaded with Calcium Sensor Dye eFluorTM 514 (eBioscience) for 30 min at 37 °C. After loading, cells were washed and resuspended in RPMI 10% FCS medium at 1 × 10^6^ ml^−1^. Anti-CD3 (OKT3, eBioscience) was added to the cells to final concentration of 0.5 μg ml^−1^. After 5 min incubation at 37 °C, a baseline measurement of 30 sec was recorded, subsequently anti-mouse IgG (Jackson ImmunoResearch) was added to a final concentration of 20 μg ml^−1^ and measurement continued for 3 min. After stimulation, 1 μg ml^−1^ of ionomycin was added to the cells and acquisition continued for 1 more min. To assess calcium influx in P3, cells were incubated in HBSS buffer containing 1 mM probenecid (Thermo Fisher) in the presence of CD3-biotin (OKT3, Biolegend) and CD28-biotin (28.2, Biolegend) or IgG2a (MOPC-173, Biolegend) antibodies for surface staining, and the calcium dye Fluo-8 AM (abcam). After 5 min incubation at 37 °C, a baseline measurement of 30 sec was recorded, streptavidin (abcam) was added and measurement continued. Following antibodies were used for surface staining: from Biolegend: CD3-BV711 (RPA-T8), CD4-BV785 (OKT4), CD45RA-PECy7 (HI100); from BD Biosciences: CD27-APC (M-T271); from Invitrogen; CD3-Qdot605 (UCHT1).

### Cell proliferation

Cells were stained for 10 min with CFSE or VPD-450 violet proliferation dye^65^, washed in PBS and cultured in growth media. After 4 days, dye dilution traces of proliferated cells were compared by flow cytometry.

### Analysis of CTLA-4 mechanisms

For CTLA-4 cycling experiments (depicted in schematic Fig. 3b), CD4 T cells were isolated from PBMCs and left to recover at least 2 h in complete RPMI media at 37 °C in 5% CO_2_ atmosphere before proceeding. Cells were then seeded to a 96-well U-bottom plate at a density of 2 × 10^6^ cells/mL and left unstimulated or stimulated with anti-CD3/CD28 coated dynabeads. After 16 h incubation, antibodies were added to stain for cycling CTLA-4 according to the following procedure: Anti-CTLA-4-PE (14D3, eBioscience) was added at 37 °C for the indicated time points (60 min, 30 min or 10 min, respectively). At time point zero, cells were placed immediately on ice and stained for surface CTLA-4. T-cell surface stains were added to all wells (anti-CD4-PerCpCy5.5 (RPA-T4, eBioscience), anti-CD25-BV605 (2A3, BD Horizon), anti-CD45RA-AF700 (HI100, BD Pharmigen)) were carried out for 30 min on ice. Cells were then washed in PBS and fixed for 1 h using the FOXP3 fixation/permeabilization kit (eBioscience). After washing in permeabilization buffer (eBioscience), intracellular stains were added for 1 h on ice. Cells were washed and analyzed by flow cytometry. Gating for naïve and memory T_regs_ and T_conv_ cells was performed as shown in Fig. S3d. Using FlowJo software (10.4), percent-quartiles as well as geometric mean fluorescence intensity values for CTLA-4-positive and FOXP3-positive or –negative final gates were extracted and normalized sample-internally to respective total CTLA-4 stains. Experiments were performed at minimum two independent blood donations of both P1 and P3.

Re-cycling CTLA-4 (depicted in schematic Fig. S3g) was analyzed as follows: Isolated CD4 T cells were stimulated for 16 h as described. In step 1, unconjugated anti-CTLA-4 antibody (Ticilimumab) was added and incubated for 60 min at 37 °C to ensure binding to the cycling portion of CTLA-4. After washing, goat-anti-human-Fc antibody (Lifetech) was then added and left to incubate for another 60 min at 37 °C, 5% CO_2_, to fluorescently label all CTLA-4 that was labelled in step 1 that had re-cycled back via the cell surface during incubation with the secondary antibody. Cells were then harvested and stained for T-cell markers. To avoid stoichiometric hindrance, cells were counter-stained for total CTLA-4 with AF647-conjugated C-19 antibody (Santa Cruz) binding the intracellular portion.

Analysis of soluble ligand uptake by memory T_regs_ was done as described previously^37^. In detail, CD4 T cells were isolated and stimulated with anti-CD3/CD28 coated dynabeads (see above), or left unstimulated. Where applicable, CD80-Ig was added and cells were incubated for 16 h. Subsequently, cells were labelled for T-cell surface markers, fixed and permeabilized as described above, and stained intracellularly with anti-CTLA-4-AF488 antibody (15162 S; Cell Signaling). CD80-Ig was visualized with anti-human IgG-PE antibody (6140–09, Southern Biotech). Where indicated, ligand binding was blocked with anti-CTLA-4 antibody (550405, BD Biosciences) during incubation. For total-uptake controls, respective samples were incubated with CD80-Ig after cell permeabilization. Using FlowJo software, cells were gated on memory T_regs_, and fluorescent values for CTLA-4 and CD80 were exported and plotted with GraphPad Prism software. Slopes of best-fit lines were calculated and compared to respective “no ligand” samples.

For analysis of transendocytosis, isolated CD4 T cells were stimulated for 16 h or left unstimulated as described above, and co-cultured with CD80-GFP expressing CHO cells. To block transendocytosis, anti-CTLA-4 (as above) was added to control conditions. Cells were then harvested and stained for T-cell markers and total CTLA-4, as described above. Populations were gated on CD4^+^ cells and plotted for CTLA-4 and transendocytosed GFP (CD80). Percent quartiles are depicted, indicating CTLA-4^+^GFP^+^ populations having transendocytosed CD80 from CHO cells and incorporated (bound to CTLA-4) into T cells.

For analysis of CTLA-4 mobilization, cells were stimulated for 60 min with PMA (20 ng/ml) and ionomycin (1 µM) in the presence of CTLA-4 antibody (as above), and subsequently stained for T-cell markers on ice. Cells were gated as shown in Fig. S4a. For reconstitution experiments, PBMCs were transfected for 24 h with pcDNA-GFP-DEF6 or pcDNA-GFP-DEF6^E331K^ using the Amaxa Nucleofector kit for primary human T cells, according to the manufacturer’s recommendations. Transfection efficiency was around 10% of GFP-expressing CD3^+^CD4^+^CD8^−^ T cells, and transfected cells were analyzed for the CTLA-4-positive portion.

Cycling assays in Jurkat cells were performed similar to respective studies on primary cells. Jurkat cells carrying mCherry-CTLA-4 were constructed by retroviral transduction. For CTLA-4 cycling, cells were stimulated o/n with OKT3, before cycling CTLA-4-BV421 antibody was added for 10 or 30 min incubation, respectively. Cells were subsequently placed on ice, and then fixed with ICFix solution (eBioscience). Rescue and overexpression experiments were performed as above, by transfecting cells 24 h prior to analysis. Gating as in Fig. S4b.

### Suppression assay

PBMCs were isolated of one healthy donor, and split into two fractions. One fraction was used for CD4 T-cell isolation by negative selection. Isolated CD4 T cells were labelled with VPD450 dye, and the following cell ratios were seeded: Respective Jurkat: unlabeled PBMCs: VPD450-labelled CD4 T cells (same healthy donor) = 2:1:1. Cells were stimulated with OKT3 and monitored at various time points by Flow cytometry. Suppression of CD4 T-cell proliferation was assessed by tracing VPD450 dye dilution. Gating as in Fig. S4d.

### Immunofluorescence

PBMCs or expanded T cells were either stimulated with antibodies against CD3/CD28 or left untreated. After a stimulation period of 48 h, cells were harvested and adhered to poly-L-lysine (Sigma)-coated cover slips by incubating cells for 10 min at 37 °C, 5% CO_2_. Cells were immediately fixed in 4% paraformaldehyde (PFA) solution. Permeabilization was done by incubation with 0.5% Triton-X100 (Sigma). Cells were blocked with 4% BSA (Roth). For staining the following antibodies were used: DEF6 (H00050619-B01, Abnova; or DEF6-antisera^20^), CTLA-4 (15162 S; Cell Signaling; or sc-376016AF647; Santa Cruz), RAB11 (sc-6565; Santa Cruz), anti-mouse (A-11029, Life Technologies), anti-rabbit (A-21429, Life Technologies), and anti-goat (Life Technologies). Cells were counterstained with 4′,6-diamidino-2-phenylindole (DAPI, Roth) for 10 mins. Coverslips were mounted in Prolong® Gold antifade reagent (Life Technologies).

### Confocal microscopy

Confocal microscopy was performed on a Leica SP8, Olympus FluoView FV10i or Zeiss LSM700 laser scanning confocal microscope, equipped with an X63 or an X40 oil lens. For the evaluation of immunological synapse formation, cell conjugates of primary NK cells and K562 cells were formed, stained and assessed by confocal microscopy^65^. For RAB11-CTLA-4-DEF6 co-localization, line scan analysis and co-localization analysis by Pearson’s correlation calculations (JaCoP tool) were performed with ImageJ^66^. Quantification of RAB11-CTLA-4 co-localization was done on areas of cells with detectable RAB11-positive vesicles and maximal CTLA-4 expression. The thickness of slices was set to 0.4 µm. For P3, images were captured on a Zeiss LSM700 microscope using a pinhole of 1 AU in each channel.

### Molecular cloning

*DEF6* and *RAB11* cloning plasmids were bought from DNASU^67^ and further subcloned into expression vectors pcDNA-GFP or pTO-STREP-HA. Mutagenesis was achieved with the Q5® Site-Directed Mutagenesis Kit (New England Biolabs) according to manufactor’s instructions. Plasmid sequences were verified through capillary sequencing.

### CRISPR/Cas9 knockout generation

sgRNA targeting exon 1 of *DEF6* (5′-ACTTGAGCAGTTCCTTGCGC-3′) or Renilla control (5′-GGTATAATACACCGCGCTAC-3′) were cloned into lentiCRISPR_v.2 plasmid, according to Zhang lab protocols (https://www.addgene.org/crispr/zhang/). Lentivirus was generated from HEK293T cells transfected with respective plasmids by calcium chloride precipitation, as described above. 48 h after transfection, virus was harvested and Jurkat-mCherry-CTLA-4 target cells (generated by similar transduction from a retroviral mCherry-CTLA-4 plasmid) were transduced by spinfection. Puromycin selection (1 ng µL^−1^) was initiated the day after, for 8 days. From batch cultures, single-cell dilutions were seeded and proliferating clones were selected and subjected to TIDE gDNA comparison and Western blot, for evaluation of knockout efficiency. gDNA was compared by Sanger sequencing with a guide-covering primer (5′-CCCCAGTGTTCGCTGATTCT-3′). TIDE sequence analysis (http://tide.deskgen.com/) of *DEF6* knockout versus Renilla control revealed over 80% editing efficiency at insertion position + 1 after Cas9 cutting site for *DEF6*, with no obvious non-edited sequences detected.

### Co-immunoprecipitation

Co-immunoprecipitation of Strep-HA-tagged RAB11 (mutants) and GFP-tagged DEF6 (mutants) were performed as follows. HEK293T cells were transfected with DNA plasmids encoding Strep-HA- or FLAG-tagged RAB11 or variants, GFP-tagged RAB11 or DEF6 (variant), or MYC-tagged DEF6 (variant), and respectively empty vectors, by calcium chloride precipitation. After incubating for 48 h at 37 °C in 5% CO_2_, cells were lysed in RIPA buffer. Lysates were cleared by high-speed centrifugation and 2 mg of respective supernatants were incubated with StrepTactin sepharose beads (IBA), EZview anti-MYC or anti-FLAG affinity gel in IP buffer (10 mM Tris, pH 7.4, 150 mM NaCl, 0.5 mM EDTA, 1 m PMSF, PIC cocktail), rotating at 4 °C. Beads were washed three times in IP buffer, and bound proteins were resolved by SDS-PAGE. Proteins were detected by immunoblotting.

### Guanine nucleotide exchange assay

GEF activity of GST-PH-DH, GST-PH-DH^E331K^ or GST control against GST-RAB11 was assessed in exchange buffer (20 mM Tris-HCL, pH 7.5, 150 mM NaCl, 1 mM MgCl_2_, 1 mM DTT, 0.01% NP-40), for enabling incorporation of Mant-GTP (Mant-GTP triethylammonium salt, Sigma, 5 µM). EDTA (1 mM) was used as positive control. Mant-GTP was monitored at 360/440 nm (excitation/emission) on a SpectraMax spectrophotometric plate reader.

### Statistical analysis

Data were analyzed with appropriate statistical tests as indicated in respective figure legends. Unpaired t tests were two-sided, Welch’s correction was applied. Data are displayed as mean ± SD, with 95% confidence intervals (where applicable). Sample sizes and replicates are indicated in figure legends.

### Reporting summary

Further information on research design is available in the Nature Research Reporting Summary linked to this article.

## Supplementary information

Supplementary Materials

Reporting Summary

Supplementary Source Data

## Data Availability

The source data underlying subpanels of Figs. 2, 3, 5, and 6, and Supplementary Figs. S1-S5 are provided as a Supplementary Source Data file. Relevant data are available from the authors. Next generation sequencing data are deposited at the European Genome-phenome Archive (EGA) which is hosted by the EBI and the CRG, under accession IDs EGAS00001003609 (P1) and EGAS00001003618 (P3 and mother). The data are not available publicly due to restrictions for controlled access, and may be accessible through the relevant Data Access Committee via formal application at the EGA (https://ega-archive.org).
