# Publisher Correction: Human DEF6 deficiency underlies an immunodeficiency syndrome with systemic autoimmunity and aberrant CTLA-4 homeostasis

**DOI:** 10.1038/s41467-019-12454-5

**Published:** 2019-10-02

**Authors:** Nina K. Serwas, Birgit Hoeger, Rico C. Ardy, Sigrun V. Stulz, Zhenhua Sui, Nima Memaran, Marie Meeths, Ana Krolo, Özlem Yüce Petronczki, Laurène Pfajfer, Tie Z. Hou, Neil Halliday, Elisangela Santos-Valente, Artem Kalinichenko, Alan Kennedy, Emily M. Mace, Malini Mukherjee, Bianca Tesi, Anna Schrempf, Winfried F. Pickl, Joanna I. Loizou, Renate Kain, Bettina Bidmon-Fliegenschnee, Jean-Nicolas Schickel, Salomé Glauzy, Jakob Huemer, Wojciech Garncarz, Elisabeth Salzer, Iro Pierides, Ivan Bilic, Jens Thiel, Peter Priftakis, Pinaki P. Banerjee, Elisabeth Förster-Waldl, David Medgyesi, Wolf-Dietrich Huber, Jordan S. Orange, Eric Meffre, David M. Sansom, Yenan T. Bryceson, Amnon Altman, Kaan Boztug

**Affiliations:** 1 Ludwig Boltzmann Institute for Rare and Undiagnosed Diseases, Vienna, Austria; 2 CeMM Research Center for Molecular Medicine of the Austrian Academy of Sciences, Vienna, Austria; 3 St. Anna Children’s Cancer Research Institute (CCRI), Vienna, Austria; 4 Centre for Hematology and Regenerative Medicine, Department of Medicine Huddinge, Karolinska Institutet, Karolinska University Hospital Huddinge, Stockholm, Sweden; 5 Division of Cell Biology, La Jolla Institute for Allergy & Immunology, La Jolla, CA 92037 USA; 6 Department of Pediatrics and Adolescent Medicine, Medical University of Vienna, Vienna, Austria; 7 Childhood Cancer Research Unit, Department of Women’s and Children’s Health, Karolinska Institutet, Karolinska University Hospital Solna, Stockholm, Sweden; 8 Clinical Genetics Unit, Department of Molecular Medicine and Surgery, and Center for Molecular Medicine, Karolinska Institutet, Karolinska University Hospital Solna, Stockholm, Sweden; 9 Center for Pathophysiology of Toulouse Purpan, INSERM UMR1043, CNRS UMR5282, Paul Sabatier University, Toulouse, France; 10 Institute of Immunity and Transplantation, Division of Infection & Immunity, School of Life and Medical Sciences, University College London, Royal Free Hospital, Rowland Hill Street, London, NW3 2PF UK; 11 Department of Pediatrics, Baylor College of Medicine and Center for Human Immunobiology, Texas Children’s Hospital, Houston, TX 77030 USA; 12 Institute of Immunology, Center for Pathophysiology, Infectiology and Immunology, Medical University of Vienna, Vienna, Austria; 13 Clinical Institute of Pathology, Medical University of Vienna, Vienna, Austria; 14 Department of Immunobiology, Yale University School of Medicine, New Haven, CT 06511 USA; 15 Department of Rheumatology and Clinical Immunology, University Medical Center Freiburg, Freiburg, 79106 Germany; 16 Astrid Lindgren Children’s Hospital, Karolinska University Hospital Huddinge, Stockholm, Sweden; 17 Department of Pediatrics and Adolescent Medicine, Division of Neonatology, Pediatric Intensive Care and Neuropediatrics, Medical University of Vienna, Vienna, Austria; 18 St. Anna Kinderspital, Department of Pediatrics and Adolescent Medicine, Medical University of Vienna, Vienna, Austria; 19 Present Address: Department of Pathology, University of California San Francisco, San Francisco, CA USA; 20 Present Address: Centre for Paediatrics and Adoloscent Medicine, Hannover Medical School, Hannover, Germany; 21 Present Address: Columbia University Medical Center, Columbia, NY USA; 22 Present Address: Takeda (Shire), Vienna, Austria; 23 Present Address: MD Anderson Cancer Center, Houston, TX USA

**Keywords:** Genetics, Immunology, Diseases

Correction to: *Nature Communications* 10.1038/s41467-019-10812-x, Article published online 15July 2019.

In the original version of this Article, an author was missing from the author list. Winfried F. Pickl should have been included between Anna Schrempf and Joanna I. Loizou. The author has been added to the list. The author affiliation to the Institute of Immunology, Center for Pathophysiology, Infectiology and Immunology, Medical University of Vienna has been added and the contribution section has been amended to include that this author performed and analysed experiments.

In addition, Laurène Pfajfer’s affiliations were not correct. The following affiliation for this author was missing: “Center for Pathophysiology of Toulouse Purpan, INSERM UMR1043, CNRS UMR5282, Paul Sabatier University, Toulouse, France.” This affiliation has been added for this author.

All other affiliations have been renumbered accordingly. The errors have been corrected in the HTML and PDF versions of this article.

