# Supplementary material for: Human DEF6 deficiency underlies an immunodeficiency syndrome with systemic autoimmunity and aberrant CTLA-4 homeostasis
Source: Nat Commun. 2019 Jul 15;10:3106. doi: 10.1038/s41467-019-10812-x (PMC6629652; doi:10.1038/s41467-019-10812-x)
Supplement: Supplementary file 1 — Supplementary Materials [file 41467_2019_10812_MOESM1_ESM.pdf]

Supplementary Materials for

Human DEF6 deficiency underlies an immunodeficiency syndrome with systemic autoimmunity and aberrant CTLA-4 homeostasis

Serwas*, Hoeger*, et al.

* equal contribution

Supplementary Figures

Fig. S1

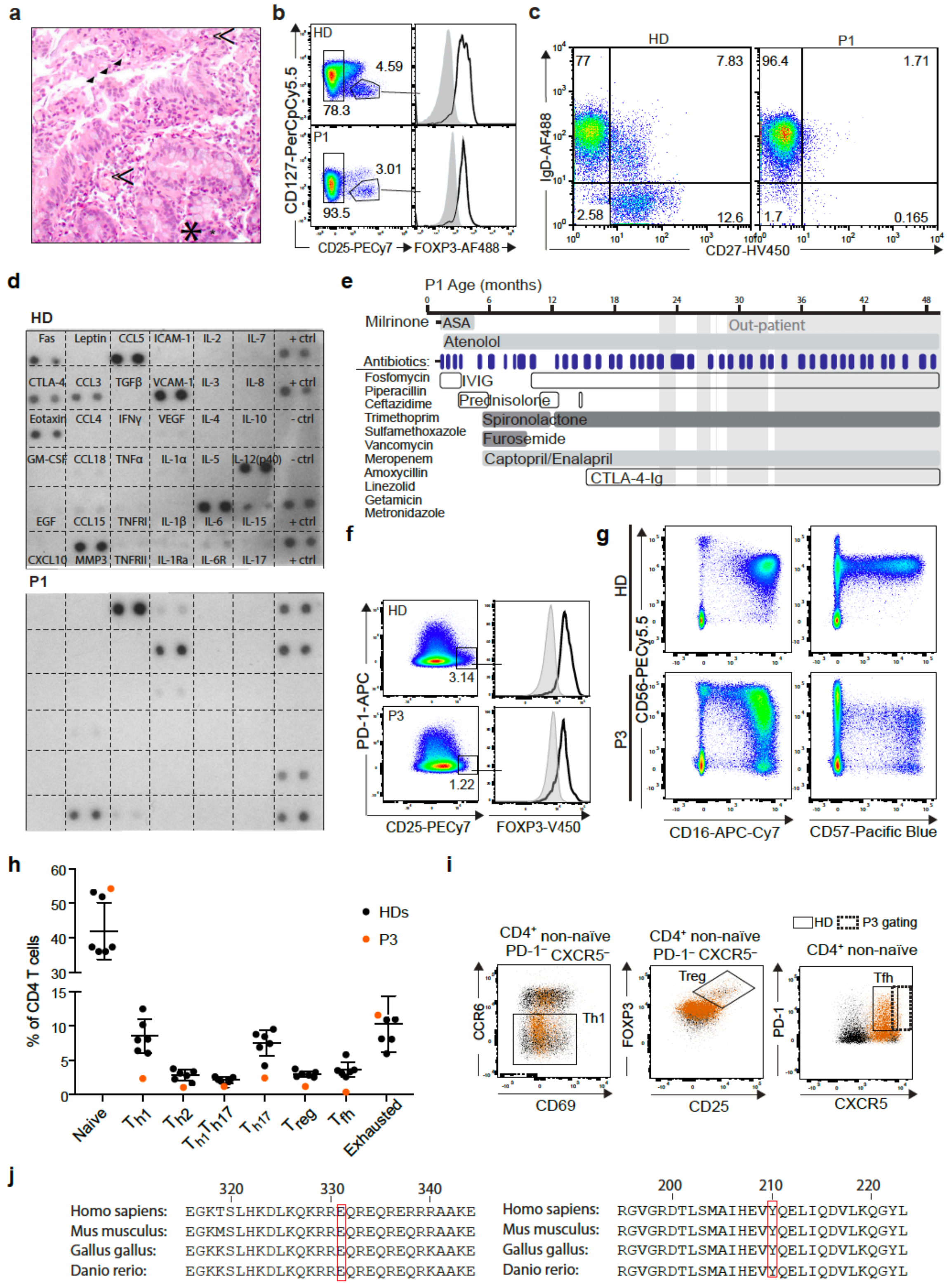

Fig. S1: Extended phenotype of the *DEF6*-mutated patients. **(a)** Duodenal biopsies of P1 at the age of 40 days showed villous atrophy (closed arrows) and an imbalance of leukocytic infiltrate with few plasma cells but increased eosinophils (lined arrows). Apoptotic bodies were visible in the epithelium of the crypts (asterisk) (H&E stain). (**b**) T_{regs} ($CD3^{+}CD4^{+}CD25^{+}CD127^{low}FOXP3^{+}$) are slightly reduced in P1, compared to HD (three donors, one representative shown). **(c)** P1 presented reduced $CD19^{+}CD27^{+}$ memory B cells, in line with her young age. **(d)** Serum cytokine blot of HD (top) and P1 (bottom) showed no systemic increase of proinflammatory cytokines. Representative of two sample shipments. **(e)** Timeline of therapeutic interventions in P1. Further medication: darbepoetin alfa, esomeprazole, cholestyramine; (gray background: out-patient; white background: in-patient (> 2 weeks)). ASA – acetyl salicylic acid. **(f)** Slightly reduced T_{regs} ($CD3^{+}CD4^{+}CXCR5^{-}PD\text{-}1^{low}CD25^{+}FOXP3^{+}$) in P3, compared to HD (representative of 6 donors). **(g)** P3 displays altered distribution of $CD3^{-}$ lymphocytes with a considerable decrease of mature ($CD56^{dim}CD16^{+}CD57^{+}$), and expanded population of $CD56^{-}CD16^{+}$ cells. **(h)** T-cell lineage phenotyping of P3 and HDs (6 donors). Naïve cells were defined as $CD3^{+}CD4^{+}CXCR5^{-}PD1^{-}CD25^{-}FOXP3^{-}CD45RA^{+}CCR7^{+}$ populations. Among non-naïve $CD4^{+}$ T cells, following populations were defined: T_{h1}: $CD25^{-}FOXP3^{-}CCR4^{-}CXCR3^{+}CCR6^{-}$, T_{h2}: $CD25^{-}FOXP3^{-}CCR4^{+}CXCR3^{-}CCR6^{-}$, T_{h17}: $CD25^{-}FOXP3^{-}CCR4^{+}CXCR3^{-}CCR6^{+}$, $T_{h1}T_{h17}$: $CD25^{-}FOXP3^{-}CCR4^{-}CXCR3^{+}CCR6^{+}$, T_{regs}: $CXCR5^{-}PD1^{-}CD25^{+}FOXP3^{+}$, T_{fh} (follicular-helper T cells): $CD25^{-}FOXP3^{-}CXCR5^{+}PD1^{+}$, Exhausted T cells: $CD25^{-}FOXP3^{-}CXCR5^{-}PD1^{+}$. Data are shown as mean ± SD. **(i)** FACS plots corresponding to (f). Gating is indicated for T_{h1}, T_{reg} and T_{fh}, with background shift in the CXCR5 staining and a slightly adapted gate for P3. A representative HD sample was

chosen. (**j**) Amino acids E331 (left) and Y210 (right) in DEF6 are conserved in vertebrates. Source data of Fig. S1 are provided as a Supplementary Source Data file.

Fig. S2

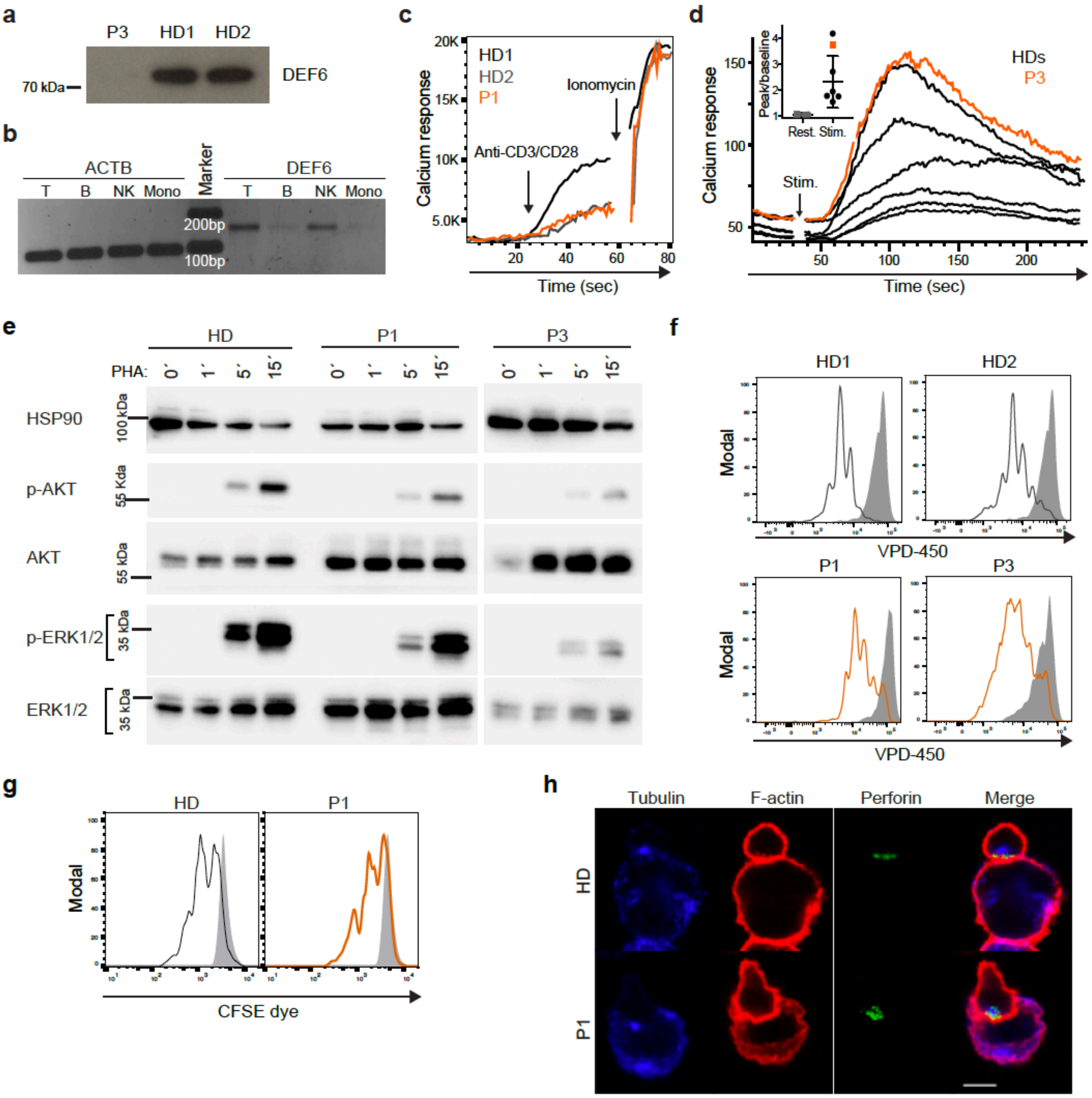

a
P3
HD1
HD2
70 kDa
DEF6
b
ACTB
T
B
NK
Mono
Marker
DEF6
200bp
100bp
c
HD1
HD2
P1
Ionomycin
Anti-CD3/CD28
Calcium response
Time (sec)
d
HDs
P3
Peak/baseline
Rest.
Stim.
Calcium response
Time (sec)
e
HD
P1
P3
PHA:
HSP90
p-AKT
AKT
p-ERK1/2
ERK1/2
100 kDa
55 Kda
55 kDa
35 kDa
35 Kda
f
HD1
HD2
P1
P3
Modal
VPD-450
g
HD
P1
Modal
CFSE dye
h
Tubulin
F-actin
Perforin
Merge
HD
P1

Fig. S2. Immunophenotypes of *DEF6*-mutant cells. **(a)** DEF6 expression in P3 and two HDs. Shorter immunoblot exposure as compared to longer exposure shown in Fig. 2e. **(b)** DEF6 is preferentially expressed in T and to a lesser extent NK cells, as analyzed by qPCR. ACTB – beta-actin control, Mono - monocytes. **(c-d)** Calcium influx in response to CD3/CD28 stimulation in primary T cells of P1 **(c)** and P3 **(d)** was in the normal range (compared to 2 (c) or 6 (d) HDs), traced by flow cytometry. **(e)** Phosphoblotting of feeder-expanded T cells after stimulation with PHA reveals slightly reduced TCR-dependent signaling in the patients, compared to healthy donor. At the indicated time points, cells were lysed and blotted for phospho-AKT, phospho-ERK and control proteins. Images were cropped for visualization. Representative of two Western blots. **(f-g)** Unaffected proliferation abilities of feeder-expanded T cells (f) and primary PBMCs (g) of patients, compared to healthy controls (HD). Cells were stimulated with anti-CD3/anti-CD28 antibodies for 5 days. Gray – non-proliferated, black – healthy donors, orange – patients. **(h)** Normal NK cell synapse formation in P1 (>12 analyzed conjugates per individual) after incubation with K562 target cells, as visualized by correct placement of perforin at the synapse edge. Scale bar – 10 µm. Source data of Fig. S2 including uncropped immunoblots are provided as a Supplementary Source Data file.

Fig. S3

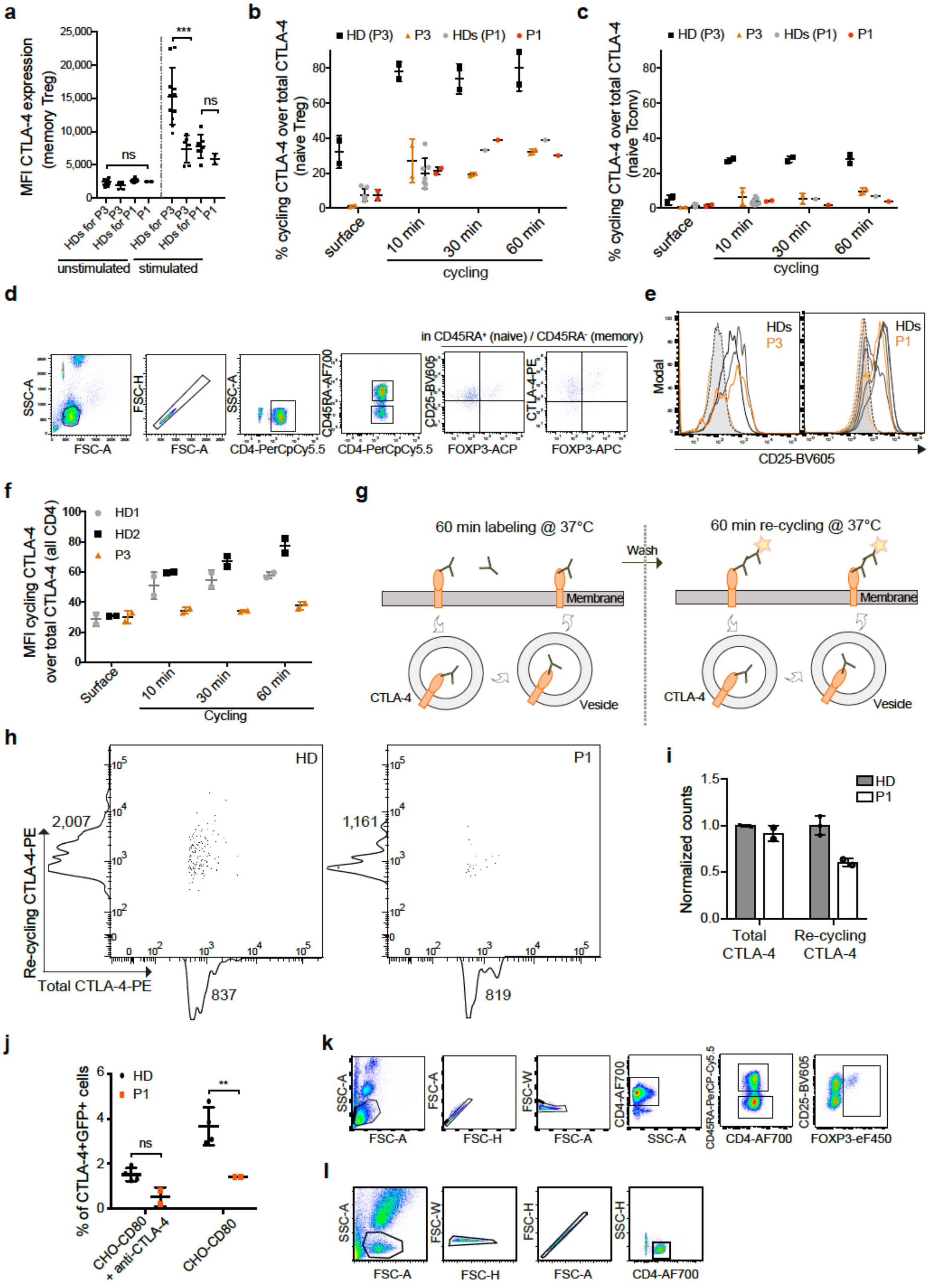

a
MFI CTLA-4 expression (memory Treg)
ns
***
ns
HDs for P3
P3
HDs for P1
P1
unstimulated
stimulated
b
HD (P3)
P3
HDs (P1)
P1
% cycling CTLA-4 over total CTLA-4 (naive Treg)
surface
10 min
30 min
60 min
cycling
c
% cycling CTLA-4 over total CTLA-4 (naive Tconv)
d
in CD45RA⁺ (naive) / CD45RA⁻ (memory)
SSC-A
FSC-A
FSC-H
CD4-PerCpCy5.5
CD45RA-AF700
CD25-BV605
FOXP3-ACP
CTLA-4-PE
FOXP3-APC
e
HDs
P3
P1
Modal
CD25-BV605
f
HD1
HD2
MFI cycling CTLA-4 over total CTLA-4 (all CD4)
Surface
Cycling
g
60 min labeling @ 37°C
Wash
60 min re-cycling @ 37°C
Membrane
CTLA-4
Vesicle
h
HD
2,007
Re-cycling CTLA-4-PE
Total CTLA-4-PE
837
1,161
819
i
Normalized counts
Total CTLA-4
Re-cycling CTLA-4
j
HD
% of CTLA-4+GFP+ cells
**
CHO-CD80 + anti-CTLA-4
CHO-CD80
k
FSC-W
CD4-AF700
SSC-A
CD45RA-PerCP-Cy5.5
FOXP3-eF450
l
SSC-H

Fig. S3. Additional evidence for the CTLA-4 trafficking defect. **(a)** Raw mean fluorescence intensity data of memory Tregs, referring to Fig. 3a. Data were overlaid with mean ± SD, pooled from up to 3 experiments. **(b-c)** Additional cycling data on P1, P3 and respective healthy controls, in naïve T_{reg} (b, $CD4^+CD45RA^+FOXP3^+$) and naïve T_{conv} cells (c, $CD4^+CD45RA^+FOXP3^-$), corresponding to Fig. 3d-e. Data were overlaid with mean ± SD. **(d)** Gating strategy for CTLA-4 cycling experiments**,** corresponding to Fig. 3c-e. **(e)** Unaffected CD25 upregulation in patient CD4 T cells. Filled gray – unstimulated; unfilled – stimulated; gray/black – HDs; orange – patients. **(f)** Defective cycling in CD4 T cells of P3, as performed for Fig. 3d-e and Fig. S3b-c, calculated for relative mean fluorescence intensities of surface/cycled CTLA-4, normalized to MFI of total CTLA-4. Data were overlaid with mean ± SD. **(g)** Schematic representation of CTLA-4 re-cycling to evaluate CTLA-4 re-appearing at outer cell membranes. **(h-i)** Reduced CTLA-4 re-cycling in memory T_{regs} of P1. Cells were stimulated with anti-CD3/anti-CD28 antibody bearing beads for 16 hours before the primary non-labeled antibody was added for 1 hour. A second antibody against the Fc portion of the primary antibody was added during the surface stain (recycling portion, see schematic shown in (g)). Total staining was performed after permeabilization. MFI values are shown (h). Cells were gated on $CD4^+CD45RA^-CD25^+FOXP3^+CTLA\text{-}4^+$ populations in isolated CD4 T cells and normalized to total CTLA-4 (i). Data are shown as mean ± SD, two independent experiments. **(j)** Quantification of two transendocytosis experiments corresponding to Fig. 4b, normalized to CTLA-4+ cells. Data were overlaid with mean ± SD. Statistics: ** p=0.008 (One-way Anova). **(k)** Gating strategy for ligand uptake experiments corresponding to Fig. 4a. **(l)** Gating strategy for transendocytosis experiments corresponding to Fig. 4b. Source data of Fig. S3 are provided as a Supplementary Source Data file.

Fig. S4

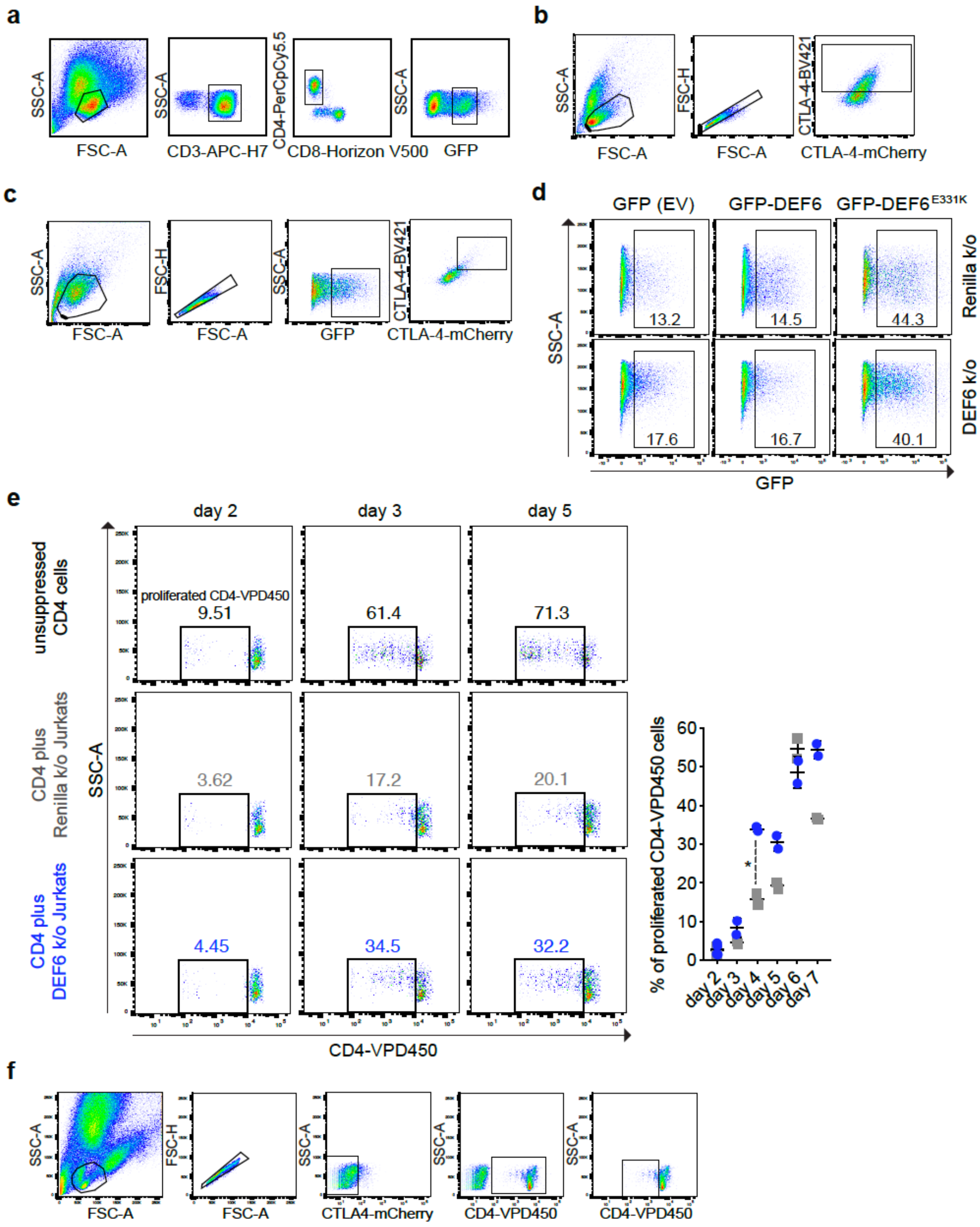
a
SSC-A
FSC-A
CD3-APC-H7
CD4-PerCpCy5.5
CD8-Horizon V500
GFP
b
FSC-H
CTLA-4-BV421
CTLA-4-mCherry
c
d
GFP (EV)
GFP-DEF6
GFP-DEF6 E331K
Renilla k/o
DEF6 k/o
13.2
14.5
44.3
17.6
16.7
40.1
e
day 2
day 3
day 5
unsuppressed CD4 cells
CD4 plus Renilla k/o Jurkats
CD4 plus DEF6 k/o Jurkats
proliferated CD4-VPD450
9.51
61.4
71.3
3.62
17.2
20.1
4.45
34.5
32.2
CD4-VPD450
% of proliferated CD4-VPD450 cells
day 2
day 3
day 4
day 5
day 6
day 7
f
CTLA4-mCherry

Fig. S4. Additional data on reconstitution and Jurkat knockouts. **(a)** Gating strategy for mobilization and reconstitution experiments in patient cells, corresponding to Fig. 5a-c. **(b)** Gating strategy (sequential) for cycling experiments on Jurkat knockout clones, corresponding to Fig. 5e. **(c)** Gating strategy for reconstitution of Jurkat knockout clones, corresponding to Fig. 5f. **(d)** GFP expression levels of Jurkat knockout clones reconstituted with GFP constructs (indicated) by electroporation, corresponding to Fig. 5f. Gating as in Fig. S4c. **(e)** DEF6 knockout in Jurkat-mCherry-CTLA-4 cells results in reduced suppression tendency against VPD450-labelled CD4 T cells compared to Renilla control, as exemplified by enhanced proliferation of VPD-labelled cells. Cells were seeded at the following ratio. Respective Jurkat : CD4 (VPD450-labelled) : PBMC (unlabeled, same healthy donor) cells = 2:1:1. VPD450 traces of proliferating CD4 T cells were followed over time. Gating as in Fig. S4f. Representative of two independent experiments. Numerical inserts show percentages of proliferated CD4 T cells. Data of both experiments are plotted to the right. Statistics: * $p=0.027$ (Unpaired t test). Data were overlaid with mean ± SD. **(f)** Gating strategy for suppression assay Fig. S4c. Source data of Fig. S4 are provided as a Supplementary Source Data file.

Fig. S5

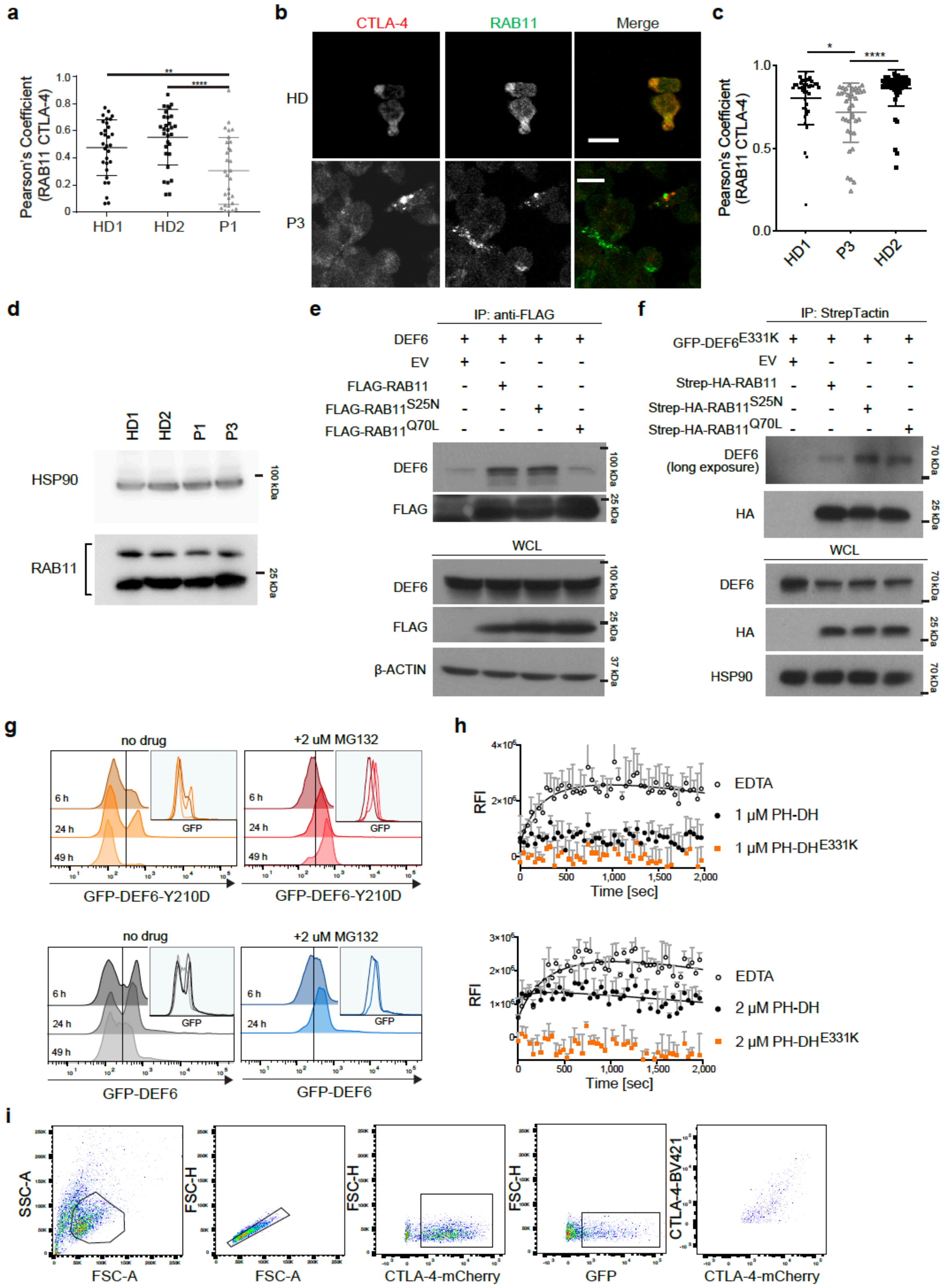

a
Pearson's Coefficient (RAB11 CTLA-4)
HD1
HD2
P1
b
CTLA-4
RAB11
Merge
HD
P3
c
Pearson's Coefficient (RAB11 CTLA-4)
HD1
P3
HD2
d
HD1
HD2
P1
P3
HSP90
RAB11
100 kDa
25 kDa
e
IP: anti-FLAG
DEF6
EV
FLAG-RAB11
FLAG-RAB11S25N
FLAG-RAB11Q70L
DEF6
FLAG
WCL
DEF6
FLAG
β-ACTIN
100 kDa
25 kDa
37 kDa
f
IP: StrepTactin
GFP-DEF6E331K
EV
Strep-HA-RAB11
Strep-HA-RAB11S25N
Strep-HA-RAB11Q70L
DEF6 (long exposure)
HA
WCL
DEF6
HA
HSP90
70 kDa
25 kDa
g
no drug
+2 uM MG132
6 h
24 h
49 h
GFP
GFP-DEF6-Y210D
GFP-DEF6
h
RFI
Time [sec]
EDTA
1 μM PH-DH
1 μM PH-DHE331K
EDTA
2 μM PH-DH
2 μM PH-DHE331K
i
SSC-A
FSC-A
FSC-H
FSC-A
FSC-H
CTLA-4-mCherry
FSC-H
GFP
CTLA-4-BV421
CTLA-4-mCherry

Fig. S5. Additional data on RAB11 and DEF6. **(a)** Quantification of RAB11/CTLA-4 co-localization after TCR stimulation in selected ROIs reveals a significant reduction in P1 compared to two HDs. Statistics: ** p=0.005; **** p<0.0001 (One-way Anova). **(b)** Reduced CTLA-4/RAB11 co-localization in P3-derived feeder-expanded stimulated T cells. Representative images of CTLA-4 and RAB11 stains in P3 and a healthy donor (shown one out of two HDs), indicating less-pronounced CTLA-4 localizing to $RAB11^{+}$ vesicles in P3. Feeder-expanded T cells were stimulated for 48h with anti-CD3/CD28 antibodies. Scale bar - 10 µm. **(c)** Quantification of CTLA-4/RAB11 co-localization in P3 corresponding to b), analyzed as for P1 (a). Statistics: * p=0.0161, **** p<0.0001 (One-way Anova). Data are shown as mean ± SD (a,c). **(d)** Feeder-expanded T cells of both patients show equal RAB11 expression as HDs. **(e-f)** Co-immunoprecipitation of RAB11 variants in HEK293T identifies DEF6 as potential GEF protein for the GTPase. Wildtype DEF6 co-immunoprecipitates with RAB11 and inactive $RAB11^{S25N}$, but not with constitutively active $RAB11^{Q70L}$ (e). $DEF6^{E331K}$ only minimally co-immunoprecipitates with either RAB11 variant (f). Long exposure times (indicated) of 30 min were necessary to visualize residual mutant DEF6 pulldowns (f, DEF6 blot, long exposure). Shorter exposure did not reveal binding (see the supplementary Source Data file). Additional HA and HSP90 blots (f) are shown in the Source Data file. Equal sample amounts were used for each experiment, sample load was balanced on immunoprecipitated RAB11. One out of two individual experiments are shown. **(g)** $DEF6^{Y210D}$ is rapidly degraded after overexpression in Jurkat cells, and can be blocked by proteasomal inhibition. GFP-tagged wildtype (bottom) or mutant (top) DEF6 were electroporated into Jurkat cells and GFP signal was traced over time, in presence or absence of MG132. **(h)** Wildtype but not mutant PH-DH shows GEF activity toward RAB11, as visualized by increased Mant-GTP intensity compared to mutant. Purified proteins were assayed, EDTA served as positive

control. RFI – relative fluorescence intensity. 1 μM (top) or 2 μM (bottom) GEF protein was used. Datapoints are shown as mean ± SD, nonlinear regression curve fitting was applied. One of two representative experiments shown. **(i)** Gating strategy for RAB11-variant cycling experiments of Fig. 6g-h. All immunoblots in this Figure were cropped for visualization. Source data of Fig. S5 including uncropped immunoblots are provided as a Supplementary Source Data file.

Supplementary Table

Table S1. Characteristics of the segregating variants in the two families.

**Gene**	**Family**	**Position***	**Ref.**	**Obs.**	**Protein**	**Poly-phen-2**	**SIFT**	**CADD**	**TOPMed allele counts****	**gnomAD allele counts**	**ExAC allele counts**	**ExAC pLI**
DEF6	Fam. A	chr6 35286024 (rs541285645)	G	A	CCDS4802; c.G991A; p.E331K	0.978	0.14	23.6	---	Hom: 0 , Het: 50 (26162) MAF: 0.001911 (SA), 0.0002674 (total)	Hom: 0, Het: 21 (8886) MAF: 0.00024 (SA)	0.98
DEF6	Fam. B	chr6 35280283	T	G	CCDS4802; c.T628G; p.Y210D	0.858	0.03	26.4	---	---	---	0.98
CR2	Fam. A	chr1 207644154 (rs781255614)	G	A	CCDS31007; c.G1295; p.G432E	1.000	0.02	25.4	Hom: 0, Het: 2 (125568) MAF: 0.000016 (total)	Hom: 0, Het: 163 (282512) MAF: 0.00057 (total)	Hom: 0, Het: 74 (16502) MAF: 0.0045 (SA)	0
SKIV2L	Fam. A	Chr6 31936274	CA	C	CCDS4731; c.C3029delA; p.Q1010fs*15	---	---	29.3	---	---	---	0

Prediction scores were calculated with Polyphen-2 (ref. [1]), SIFT[2] and CADD[3]. Variant counts (in brackets: total analyzed alleles) and frequencies were downloaded from gnomAD (https://gnomad.broadinstitute.org/) or ExAC (http://exac.broadinstitute.org/) databases[4], or the TOPMed database via the Bravo browser (https://bravo.sph.umich.edu/freeze5/hg38/). Current genomes include: gnomAD – 10,738, ExAC – 60,706, TOPMed Bravo – 62,784 individuals. MAF frequencies are indicated for identified heterozygotes. (Accession date: March 31, 2019) (Ref.: reference; Obs.: observed; het: heterozygous; hom: homozygous; MAF – minor allele frequency; SA – South Asian; pLI: probability of loss of function intolerance of homozygous/heterozygous variants; --- – not reported; * human reference genome GRCh37; ** positions were converted and extracted for GRCh38 reference genomes).

Supplementary References

1. Adzhubei, I.A. *et al.* A method and server for predicting damaging missense mutations. *Nat Methods* **7**, 248-249 (2010).

2. Kumar, P., Henikoff, S. & Ng, P.C. Predicting the effects of coding non-synonymous variants on protein function using the SIFT algorithm. *Nat Protoc* **4**, 1073-1081 (2009).

3. Kircher, M. *et al.* A general framework for estimating the relative pathogenicity of human genetic variants. *Nat Genet* **46**, 310-315 (2014).

4. Lek, M. *et al.* Analysis of protein-coding genetic variation in 60,706 humans. *Nature* **536**, 285-291 (2016).
