# Supplementary material for: Human DEF6 deficiency underlies an immunodeficiency syndrome with systemic autoimmunity and aberrant CTLA-4 homeostasis
Source: Nat Commun. 2019 Jul 15;10:3106. doi: 10.1038/s41467-019-10812-x (PMC6629652; doi:10.1038/s41467-019-10812-x)
Supplement: Supplementary file 2 — Reporting Summary [file 41467_2019_10812_MOESM2_ESM.pdf]

nature research

Corresponding author(s): Kaan Boztug, NCOMMS-18-2707B

Reporting Summary

Nature Research wishes to improve the reproducibility of the work that we publish. This form provides structure for consistency and transparency in reporting. For further information on Nature Research policies, see Authors & Referees and the Editorial Policy Checklist.

Statistical parameters

When statistical analyses are reported, confirm that the following items are present in the relevant location (e.g. figure legend, table legend, main text, or Methods section).

n/a	Confirmed	
☐	☒	The exact sample size (*n*) for each experimental group/condition, given as a discrete number and unit of measurement
☐	☒	An indication of whether measurements were taken from distinct samples or whether the same sample was measured repeatedly
☐	☒	The statistical test(s) used AND whether they are one- or two-sided<br>*Only common tests should be described solely by name; describe more complex techniques in the Methods section.*
☒	☐	A description of all covariates tested
☐	☒	A description of any assumptions or corrections, such as tests of normality and adjustment for multiple comparisons
☐	☒	A full description of the statistics including central tendency (e.g. means) or other basic estimates (e.g. regression coefficient) AND variation (e.g. standard deviation) or associated estimates of uncertainty (e.g. confidence intervals)
☐	☒	For null hypothesis testing, the test statistic (e.g. *F*, *t*, *r*) with confidence intervals, effect sizes, degrees of freedom and *P* value noted<br>*Give P values as exact values whenever suitable.*
☒	☐	For Bayesian analysis, information on the choice of priors and Markov chain Monte Carlo settings
☒	☐	For hierarchical and complex designs, identification of the appropriate level for tests and full reporting of outcomes
☐	☒	Estimates of effect sizes (e.g. Cohen's *d*, Pearson's *r*), indicating how they were calculated
☐	☒	Clearly defined error bars<br>*State explicitly what error bars represent (e.g. SD, SE, CI)*

Our web collection on statistics for biologists may be useful.

Software and code

Policy information about availability of computer code

Data collection	No code was used to collect data.
Data analysis	Software for data analysis is specified and referenced in the Methods section.

For manuscripts utilizing custom algorithms or software that are central to the research but not yet described in published literature, software must be made available to editors/reviewers upon request. We strongly encourage code deposition in a community repository (e.g. GitHub). See the Nature Research guidelines for submitting code & software for further information.

Data

Policy information about availability of data

All manuscripts must include a data availability statement. This statement should provide the following information, where applicable:

- Accession codes, unique identifiers, or web links for publicly available datasets
- A list of figures that have associated raw data
- A description of any restrictions on data availability

Data corresponding to Figs 2,3,5,6 and Supplementary Figs S1-S5 are provided in a Source Data file.

Field-specific reporting

Please select the best fit for your research. If you are not sure, read the appropriate sections before making your selection.

☒ Life sciences ☐ Behavioural & social sciences ☐ Ecological, evolutionary & environmental sciences

For a reference copy of the document with all sections, see nature.com/authors/policies/ReportingSummary-flat.pdf

Life sciences study design

All studies must disclose on these points even when the disclosure is negative.

Sample size	Sample size reflects availability of patient material.
Data exclusions	No data points were excluded from analysis.
Replication	Experiments were performed in replicates as stated in respective Figure legends.
Randomization	Patient samples were analyzed individually in comparison to healthy donor controls (indicating sample sizes in figures and figure legends)
Blinding	No investigators were blinded during data collection or analysis; patient samples were treated individually and not grouped.

Reporting for specific materials, systems and methods

Materials & experimental systems

n/a	Involved in the study
☐	☒ Unique biological materials
☐	☒ Antibodies
☐	☒ Eukaryotic cell lines
☒	☐ Palaeontology
☒	☐ Animals and other organisms
☐	☒ Human research participants

Methods

n/a	Involved in the study
☒	☐ ChIP-seq
☐	☒ Flow cytometry
☒	☐ MRI-based neuroimaging

Unique biological materials

Policy information about availability of materials

Obtaining unique materials	Patient-derived (unique) material may be accessible through the corresponding author, only upon patients' consent and allowing clinical conditions and ethical approval, upon reasonable request.

Antibodies

Antibodies used	Methods paragraphs "Flow cytometry", "Cell culture and stimulation conditions", "Immunoblots", "Phosphoblotting", "Calcium flux", "Analysis of CTLA-4 mechanisms", "Immunofluorescence", "Co-immunoprecipitation", on all antibodies and their specifications.
Validation	Respective manufacturer's validation data as provided online.

Eukaryotic cell lines

Policy information about cell lines

Cell line source(s)	CHO cells were a kind gift from David Sansom, UCL. Jurkat E6.1 and HEK293 cells were provided by the Center of Molecular Medicine.
Authentication	Authentication of cell lines (see above) was not performed after receival.
Mycoplasma contamination	All cell lines were regularly tested negative for mycoplasma contamination.

Commonly misidentified lines (See ICLAC register)	n/a

Human research participants

Policy information about studies involving human research participants

Population characteristics	No population studies were performed.
Recruitment	No population studies were performed.

Flow Cytometry

Plots

Confirm that:

- [x] The axis labels state the marker and fluorochrome used (e.g. CD4-FITC).
- [x] The axis scales are clearly visible. Include numbers along axes only for bottom left plot of group (a 'group' is an analysis of identical markers).
- [x] All plots are contour plots with outliers or pseudocolor plots.
- [x] A numerical value for number of cells or percentage (with statistics) is provided.

Methodology

Sample preparation	Methods sections "Flow cytometry", "Cell culture and stimulation", "Analysis of CTLA-4 mechanisms"
Instrument	BD LSR Fortessa (P1 and P3 data) BD FACSCanto II (P3 immunophenotyping)
Software	Data were collected with FACSDiva 8.0.1, and analyzed by FlowJo software version 9 or higher.
Cell population abundance	Cell lines were regularly confirmed for at least 90 percent of positive population, according to flow cytometry validation.
Gating strategy	Gating strategies are depicted in Supplementary Figures S3, S4, S5, and referred to in all respective Figure Legends.

- [x] Tick this box to confirm that a figure exemplifying the gating strategy is provided in the Supplementary Information.
